# Large clones of pre-existing T cells drive early immunity against SARS-COV-2 and LCMV infection

**DOI:** 10.1016/j.isci.2023.106937

**Published:** 2023-05-22

**Authors:** Martina Milighetti, Yanchun Peng, Cedric Tan, Michal Mark, Gayathri Nageswaran, Suzanne Byrne, Tahel Ronel, Tom Peacock, Andreas Mayer, Aneesh Chandran, Joshua Rosenheim, Matthew Whelan, Xuan Yao, Guihai Liu, Suet Ling Felce, Tao Dong, Alexander J. Mentzer, Julian C. Knight, Francois Balloux, Erez Greenstein, Shlomit Reich-Zeliger, Corinna Pade, Joseph M. Gibbons, Amanda Semper, Tim Brooks, Ashley Otter, Daniel M. Altmann, Rosemary J. Boyton, Mala K. Maini, Aine McKnight, Charlotte Manisty, Thomas A. Treibel, James C. Moon, COVIDsortium Investigators, Mahdad Noursadeghi, Benny Chain

**Affiliations:** 1 Division of Infection and Immunity, University College London, London WC1E 6BT, UK; 2 MRC Human Immunology Unit, MRC Weatherall Institute of Molecular Medicine, Radcliffe Department of Medicine, University of Oxford, Oxford, UK; 3 Chinese Academy of Medical Science (CAMS) Oxford Institute (COI), University of Oxford, Oxford, UK; 4 Wellcome Centre for Human Genetics, University of Oxford, Oxford, UK; 5 UCL Genetics Institute, University College London, London WC1E 6BT, UK; 6 Department of Systems Immunology, Weizmann Institute of Science, Rehovot 7610001, Israel; 7 Blizard Institute, Barts and the London School of Medicine and Dentistry, Queen Mary University of London, London E1 4NS, UK; 8 UK Health Security Agency, Porton Down, Salisbury SP4 0JG, UK; 9 Department of Immunology and Inflammation, Imperial College London, London SW7 2BX, UK; 10 Department of Infectious Disease, Imperial College London, London W12 0NN, UK; 11 Lung Division, Royal Brompton Hospital, Guy’s and St Thomas’ NHS Foundation Trust, London, UK; 12 Institute of Cardiovascular Sciences, University College London, London WC1E 6BT, UK

**Keywords:** Biological sciences, Immunology, Immunity, Cell biology

## Abstract

T cell responses precede antibody and may provide early control of infection. We analyzed the clonal basis of this rapid response following SARS-COV-2 infection. We applied T cell receptor (TCR) sequencing to define the trajectories of individual T cell clones immediately. In SARS-COV-2 PCR+ individuals, a wave of TCRs strongly but transiently expand, frequently peaking the same week as the first positive PCR test. These expanding TCR CDR3s were enriched for sequences functionally annotated as SARS-COV-2 specific. Epitopes recognized by the expanding TCRs were highly conserved between SARS-COV-2 strains but not with circulating human coronaviruses. Many expanding CDR3s were present at high frequency in pre-pandemic repertoires. Early response TCRs specific for lymphocytic choriomeningitis virus epitopes were also found at high frequency in the preinfection naive repertoire. High-frequency naive precursors may allow the T cell response to respond rapidly during the crucial early phases of acute viral infection.

## Highlights

• SARS-COV-2 infection drives a rapid wave of T cell clonal expansion
• Epitopes recognized by early expanding TCRs are highly conserved between strains
• Early expanding T cell receptors found at high frequency in pre-exposure repertoires
• High-frequency naive T cell clones may provide early protection to viral infection

## Graphical abstract

**Figure:**
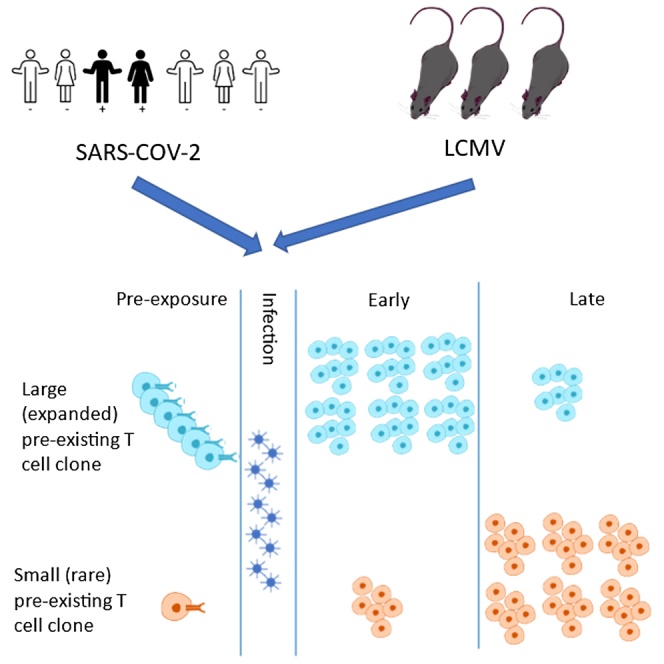

## Introduction

T cell responses to vaccination^1^ and acute infection^2^ are often fast, with the magnitude of the response peaking within one to two weeks of exposure and substantially preceding maximum antibody responses. T cells may therefore combine with innate immunity to control microbial growth early in infection, before humoral immunity is fully developed. The mechanisms which underly this early clonal expansion remain poorly defined. In this study, we exploit a granular longitudinal sampling of peripheral blood cells which were collected during, and in some cases preceding, acute infection with SARS-COV-2, during the first few weeks of the pandemic.^3^ The dynamic changes in the T cell receptor repertoire which are observed during this period reveal mechanisms that allow the speed of the T cell adaptive response to compete with that of innate immunity and viral replication during the crucial early phases of this acute viral infection.

There is an increasing interest in the role of T cells in providing protection against SARS-COV-2 infection and disease.^4^ HLA-peptide binding and *in vitro* peptide-driven expansion have provided a comprehensive catalog of T cell epitopes,^5^^,^^6^ which span most of the viral open reading frames (ORFs). Single cell protein and transcriptomic analyses have provided complementary information on memory and effector T cell function.^7^^,^^8^ There have also been many attempts to link T cell responses to clinical outcome, although distinguishing cause and effect in these observational studies is challenging, especially because SARS-COV-2 disease is often associated with lymphopoenia. Nevertheless, there is some evidence that T cell responses may be associated with better clinical outcomes^9^^,^^10^^,^^11^ and this has fuelled the development of more T cell centric vaccines, especially in individuals with impaired humoral immunity.^12^

Despite the wealth of published studies on SARS-COV-2, however, dynamic analysis of immune responses in the early phases of infection are still rare.^13^ Indeed, detailed dynamics of the earliest responses are rare in any human infection. Experimental infection studies (e.g., to influenza) have provided some important data in this regard, but prior exposure is often a confounder in this setting.^14^ We have previously reported on the COVIDSortium,^15^ a longitudinal study of London-based health care workers, established very early in the first wave of the COVID pandemic, which provides a detailed granular view of the peripheral blood compartment during the first few weeks of infection.^3^ We have used this sample set to demonstrate a remarkably fast CD4 and CD8 T cell response following SARS-COV-2 infection, which parallels the innate interferon response, and substantially precedes humoral immunity.^15^ The appearance of high-frequency (>100 TCR alpha/beta per million) T cells in the blood at the time of the first positive SARS-COV-2 PCR, or in some cases even before PCR+, needs to be considered in the context of the T cell precursor frequency prior to infection, and the known dynamics of T cell proliferation. The length of time between initial exposure and detectable SARS-COV-2 viremia (incubation period) has been estimated to be between 4 and 6 days.^16^^,^^17^^,^^18^^,^^19^ There is a lag between exposure and the arrival of antigen in the draining lymph node, and a further lag between TCR binding and the first cycle of T cell proliferation. Finally the time taken for a human T cell to complete the cell cycle is in the order of 12–14 h (Phil Hodgkin, personal communication). There is therefore time for no more than a maximum of 10 T cell divisions which, without taking into account cell death, allows each cell to proliferate 2^10^ (approximately 1,000-fold increase). The median frequency of the expanded TCR alpha and beta receptors prior to expansion must therefore be at least in the order of 1 in 10^7^. In a total pool of 10^11^ naive T cells, this corresponds to a large (10,000) precursor T cell clone size.

In the present study, we investigate in more detail clonal expansions which contribute to this early T cell response, using robust quantitative T cell repertoire sequencing to chart the evolution of the anti-viral response at the level of the clonotype.

Global TCRseq provides an unbiased survey of the T cell response, which is complementary to more antigen-specific methodologies which depend on predefined targets such as peptides or HLA multimers. We identify a wave of early T cells, which are enriched for sequences independently annotated as SARS-CoV-2-specific. Many of the CDR3 sequences are found in the pre-pandemic repertoires of healthy individuals, and we leverage this information to develop a statistical framework to infer that they are present at high clonal frequency in pre-pandemic repertoires. Remarkably, we extend these observations to similar early expanding epitope-specific T cell receptors (TCRs) in LCMV infection, and demonstrate that many of these sequences can be found at high abundance in naive repertoires prior to and independently of exposure to lymphocytic choriomeningitis virus (LCMV). The ability to rapidly recruit high abundance TCRs commonly found in the naive TCR repertoire may be a common strategy to cope with the response to novel microbial pathogens.

## Results

### An early transient wave of TCR expansion associated with infection with Sars-Cov-2

We sequenced the T cell repertoire of whole blood RNA samples collected at different time points (see below) from 41 health care workers (HCW) who tested PCR+ for SARS-COV-2 infection, and 6 HCW who remained PCR and seronegative throughout the study (sample collection summarized in Figure S1). The samples were collected at weeks 0–4 (acute) and week 12–14 (convalescent). The median number of samples collected for each individual was 4, ranging from 1 to 6. The majority of infections registered a PCR+ test at the first sample, but in 12 individuals, samples predating the first PCR+ sample were obtained. The first 3–4 weeks of sample collection coincided with the first wave of the SARS-COV-2 pandemic in London, and no additional cases of PCR+ confirmed infection or seroconversion were observed in the subsequent 2–3 months of collection. We sequenced a total of 14.6 million TCR alpha and TCR beta genes, with a median 73,000 TCR alpha and 94,000 TCR beta sequences per sample (Figure S2A). The number of alpha and beta sequences per sample were highly correlated (Spearman correlation = 0.93, Figure S2B), providing additional confidence in the quantitative robustness of the pipeline.

We first measured the richness (defined as number of distinct sequences, divided by total number of sequences in sample) and Shannon diversity (which incorporates a measure of the frequency distribution of the sequences) of the repertoires at different time points in both PCR+ and PCR-individuals. For PCR+ individuals we plotted the values relative to the week at which the individual first became PCR+ (Figure S3). However, there were no detectable effects of infection on the global parameters of the TCR repertoire.

**Figure 1:**
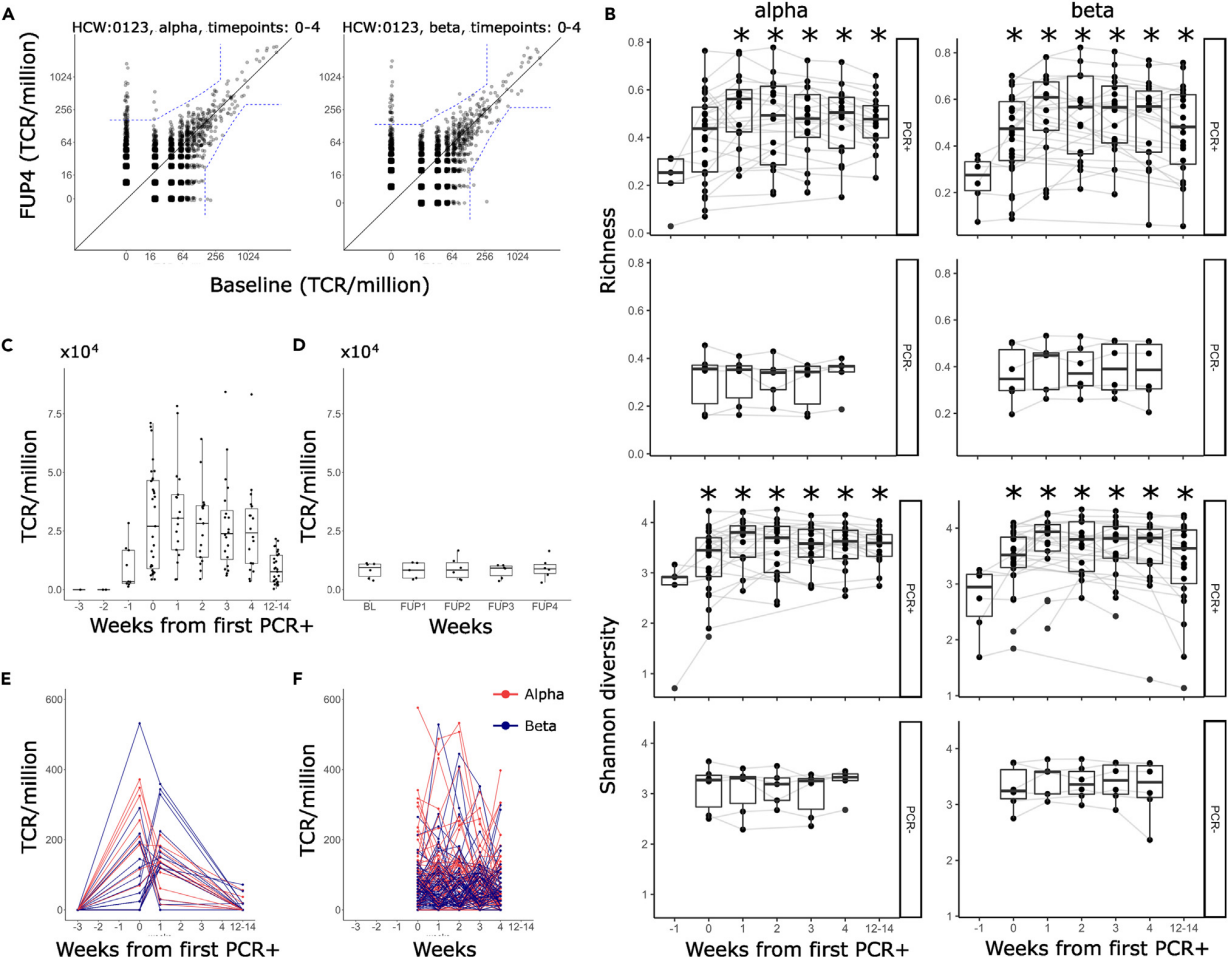
An early transient wave of TCR expansion associated with infection with SARS-COV-2 (A) An example of a pairwise comparison between two time points, showing TCRs expanding between baseline and follow-up week 4 (FUP4) repertoires. The individual (ID 123) became PCR+ at follow-up week 1 (FUP1). Each point is an individual TCR sequence, plotting its abundance at FUP4 versus its abundance at Baseline. All abundances are normalized to number of TCRs per million. The dashed blue line indicates the significance threshold calculated as described in M&M. All TCRs which fall above and to the left of the dashed line are considered as expanded while all those which fall below and to the right are contracted. (B) The richness and Shannon diversity of the set of expanded or contracted TCRs at each time point, subsampled to similar dataset size. For PCR+ individuals, the x axis is rescaled relative to the week at which they first became PCR+ (this is week 0). For PCR-individuals the weeks correspond to baseline and subsequent follow-ups at weeks 1–4. The boxplots show median, interquartile (box) and 95% (whiskers) range. ∗p value <0.05, Wilcox test compared to week −1. (C) The sum of the expanded TCRs at each time point, for each infected individual. The timepoints have been recalibrated relative to the week of the first PCR+ test (this is defined as week 0). The boxplots show median, interquartile (box) and 95% (whiskers) range. (D) As for C, but for controls who did not become PCR+. (E) The dynamics of individual TCRs (TCRalpha in red, TCRbeta in blue) in one individual (ID 101). (F) As for E but for a PCR- (ID 17). The dynamics for each individual HCW are shown in supplementary data.

We therefore focused on identifying individual TCRs which changed significantly in abundance within the first 5 weeks of the study (see STAR Methods and^15^ for further details). In some pairwise comparisons a clear population of expanded TCRs was observed (Figure 1A). In others, especially where the first sample available was already PCR+, a population of contracting TCRs was observed (Figure S4A). In these cases, we made the assumption that we had missed the expansion phase but observed the contraction phase of the initial T cell response. We therefore combined all up and downregulated TCRs, which we refer to as expanded TCRs, and removed MAIT cells (defined as TRAV1-2 paired with TRAJ12, TRAJ20, or TRAJ33) and iNKT cells (identified as TRAV10 paired with TRAJ18) from further analysis, leaving 4,075 expanded TCR alpha sequences and 5,458 expanded TCR beta sequences. In control uninfected individuals there were few expanding or contracting TCRs (Figure S4B). The richness and diversity of the set of expanded TCRs increased at the time of first PCR+ in infected individuals (p value <0.05 for all time points compared to pre-infection, Wilcoxon signed-rank test) but showed no consistent dynamics in uninfected individuals (Figure 1B). Note that the time axis was rescaled relative to the week at which that individual became PCR+ (renamed as week 0).

We plotted the combined abundances of all expanded TCRs for each individual (an estimate of total clonal expansion) as a function of time (Figure 1C). The magnitude of the response varied considerably between individuals but showed a common dynamic. The response was already strong at week 0 and fell by week 14. In some individuals responses could be detected even in the week prior to becoming PCR+. The magnitude of the response of the “expanded” TCRs in the PCR-seronegative controls showed no evidence of any common dynamic pattern (Figure 1D). For comparison we show the dynamics of the anti-spike (Figure S5A) and anti-nuclear protein (Figure S5B) antibody titers, which rose more gradually and slowly over the first 14 weeks, as described in detail previously.^20^

An example of the time course of an individual HCW, for whom we had samples pre- as well as post-infection, shows more clearly the rapid expansion of individual TCR alpha and TCR beta sequences, which were mostly not detected pre-infection, because of sampling only a very small proportion of the repertoire but maximal at either week 0 (week first PCR+) or week 1 (Figure 1E). Although the magnitude of the T cell response returns toward baseline by week 14, the expanded TCRs in this individual remain significantly above their starting abundance (Figure S6). For comparison, the timecourse of TCRs in an individual who did not become PCR+ show no clear pattern over the weeks of sampling (Figure 1F). The individual time courses for TCRs for all HCW are shown in Figure S7.

In summary, analysis of the dynamics of the TCR repertoire over the first 4 weeks identified a set of TCRs whose abundance increased rapidly and transitorily around or shortly after the time of SARS-COV-2 detection.

### Functional annotation for SARS-COV-2 and sequence similarity characterize the early wave of expanded TCRs in PCR+ individuals

**Figure 2:**
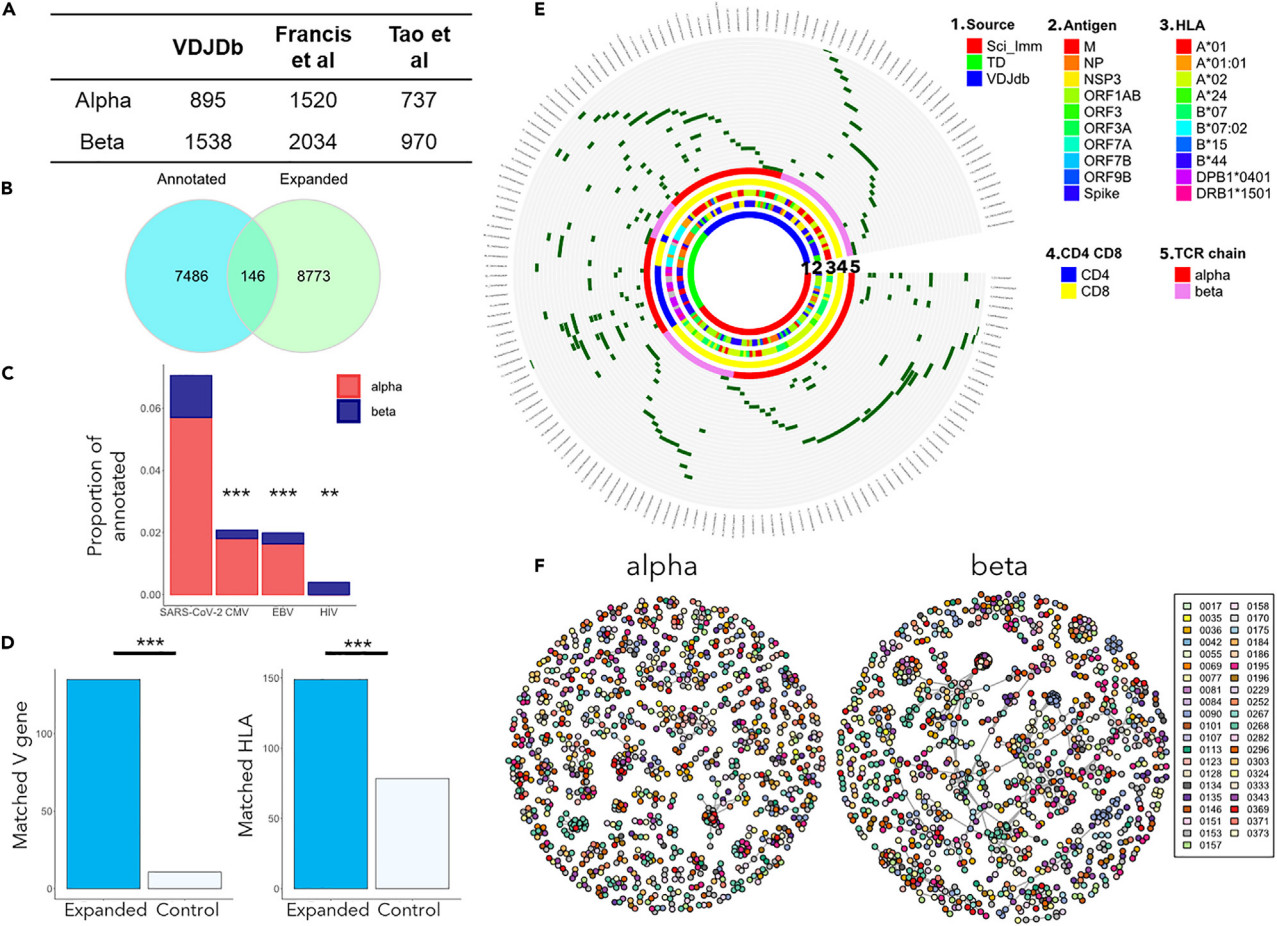
Functional annotation for SARS-COV-2 and sequence similarity characterize the early wave of expanded TCRs in PCR+ individuals (A) We collected a set of TCR sequences which had been functionally annotated for recognition of SARS-COV-2 epitopes. The set was compiled from three datasets.^21^^,^^22^^,^^23^ The set combines paired alpha/beta sequences and unpaired sequences. The Table shows the number of alpha and beta sequences in each set. (B) The intersection between the expanded TCRs as defined in Figure 1 (green), and the SARS-COV-2 annotated TCRs as in panel A (blue). (C) The proportion of annotated TCRs for each of four viruses which are observed in the expanded set of TCRs defined in Figure 1. SARS-COV-2 annotated TCRs are found significantly more often than those for CMV, EBV and HIV (∗∗∗p < 0.0001, ∗∗p < 0.001, Fisher’s exact test). (D) Left panel. Blue shading the number of expanded TCR CDR3s which share the same V gene as the respective identical annotated TCR CDR3. Empty bars – the number of matches after random shuffling of the expanded set with respect to their V gene. Right panel. Blue shading – the number of annotated TCRs whose restricting HLA gene matches at least one allele of the individual in whom the identical expanded CDR3 is detected. Empty bars – the number of matched TCRs after random shuffling of the annotated TCRs with respect to their HLA restriction (∗∗∗p < 0.0001, Fisher’s exact test). (E) A circus plot showing distribution of the annotated expanded TCRs in the cohort, and the metadata associated with each TCR. Each segment is a unique CDR3. Each circle is a different individual. Dark green segments correspond to an annotated expanded TCR. The inner circles show which CDR3 are alpha or beta; which CDR3 are derived from CD4 or CD8 T cells; the restricting HLA allele for each CDR3; the target antigen recognized by the annotated TCR; and the source of the CDR3. (F) A graph representation of the TCR alpha and beta sequence similarity. Each node is a CDR3 sequence, and edges connect all nodes with a string kernel similarity index of greater than 0.76 For TCRalpha and 0.72 TCRbeta. Each individual is shown in a different color. Only clusters of three or more connected nodes are shown.

We collected a set of 7,694 (7,632 unique across all sets, Figure 2A) functionally annotated TCRs, combining from a public TCR database VDJDb,^21^ a published study^22^ and a set of TCRs we identified by tetramer sorting or *in vitro* peptide expansions.^23^ The functional affinity of the TCRs varied over a broad range but included several high affinity clones (Figure S8). 239 (141 unique) CDR3s from the expanded TCR set matched with CDR3 sequences in the annotated TCR sequences (3.1%, Figure 2B). The majority of matches were with alpha chains. Same size random sets of non-expanded TCRs from the HCW repertoires matched an average of 4.2 (0.06%) annotated TCRs, significantly (more than 10-fold) less than the number of annotated sequences in the expanded set (p < 0.0001, Fisher’s exact test). There were only 7 matches of SARS-COV-2 annotated CDR3 with the set of expanded TCRs from the control (PCR-) repertoires.

We examined whether the expanded sets were also enriched for CMV annotated or EBV annotated TCR, since these viruses are prevalent in the general population (Figure 2C). The enrichment for SARS-COV-2 annotated TCRs was significantly higher than for CMV or EBV. As a comparison, we looked for matches with HIV annotated TCRs, since this virus is not likely to be prevalent in the general population. The proportion of matches with the HIV set was smaller than CMV or EBV, but the lack of annotated HIV-specific TCRalpha sequences in the database complicates interpretation.

Sharing of an identical CDR3 sequence between SARS-COV-2 annotated TCRs and the expanded set of TCRs identified in this study does not necessarily guarantee that the antigen target of the CDR3s is the same. However, we observed that the expanded CDR3 in our data was associated with the same TCR V gene as reported in the annotated set (Figure 2D, left). In addition, the HLA of the individuals in whom we observed expanded matched CDR3s was associated with the HLA restriction reported for the epitope reported for each annotated TCR (Figure 2D, right). The sharing of CDR3 sequence, V gene and HLA restriction are together suggestive of shared epitope recognition.

The distribution of annotated expanded TCRs among the PCR+ HCW cohort, and the associated metadata available for each epitope are shown in Figure 2E. Almost every PCR+ individual contained at least one annotated expanded TCR, and many contained several. The annotated matched TCRs included both CD4^+^ and CD8^+^ cells, were restricted by a variety of HLA alleles and recognized epitopes from a variety of structural and non-structural viral proteins. The time course of the annotated TCRs broadly matched that of the total set of expanded TCRs, with the majority of TCRs peaking the week of the first PCR+ test (Figure S9).

A number of expanded TCRs (including some expanded annotated TCRs, Figure 2D) were found in more than one individual. Since TCRs with similar CDR3 sequence often recognize the same antigen^24^^,^^25^^,^^26^ we looked for sharing of similar, as well as identical expanded TCRs between individuals. Similarity was measured using a triplet kernel as described previously^27^ and in M&M. We observed that a substantial proportion of the expanded TCRs formed inter-individual clusters of similar sequence, indicating that similar TCRs were frequently expanded in different individuals following SARS-COV-2 infection (Figure 2F). The proportion of control (non-expanded) TCRs forming clusters was much less (Figure S10). Some of the clustered expanded TCR sequences were also functionally annotated, and where more than one annotated TCR was found within a cluster, the target antigen was generally the same (Figure S11).

Taken together, the data presented in Figure 2 support the hypothesis that the early wave of expanding TCRs which follow infection with SARS-COV-2 represent a set of virus specific TCRs, many of which share similar or identical sequences in different individuals.

### Expanding TCRs associated with SARS-COV-2 infection are abundant in healthy pre-pandemic repertoires

**Figure 3:**
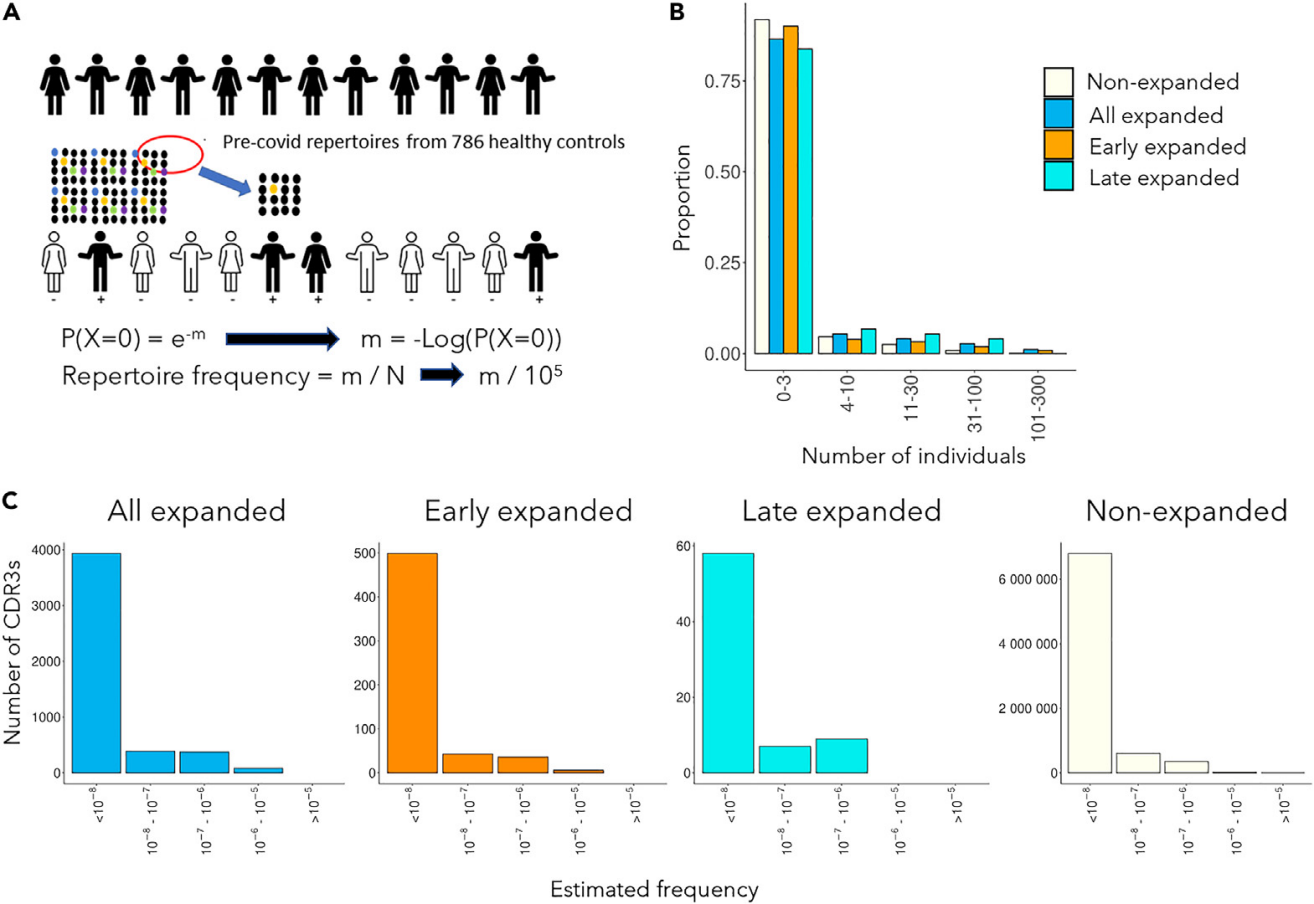
Expanding TCRs associated with SARS-COV-2 infection are abundant in healthy pre-pandemic repertoires (A) Schematic of statistical inference of TCR frequencies from abundance data in pre-pandemic repertoires. m is the mean number of times a particular TCR is present in a given sample, given that that TCR is NOT detected in a proportion P of the 786 pre-pandemic repertoires examined. The estimated frequency is given by m/N, where N is the average number of TCRs sequenced in each sample. (B) Sharing of expanded TCRbeta or non-expanded control TCRs across the 786 repertoires of the Emerson dataset, collected and sequenced several years before the SARS-CoV-2 pandemic. The x axis shows the number of individuals each TCR is observed in. The y axis shows the proportion of the expanded TCRs with a given sharing level. The expanded set is shown both as a whole, as well as split into early and late, as defined in Figure S13. (C) The estimated frequency distribution of the 2648 expanded TCR beta sequences, and a same size set of non-expanded TCRs which are found in 2 or more individuals of the Emerson dataset. The frequency of each expanded TCR was estimated using Equation 2, as discussed in the text. TCR which were found once or zero times in the Emerson data were assigned a frequency of <10^−6^and are represented by the column closest to the y axis.

The peak in TCR expansion observed around the time of PCR+ virus detection suggests a very rapid T cell response. We explored the possibility that the rapid expansion of TCRs arises from sequences which are present at higher than average frequency in the pre-infection repertoire. We developed a statistical framework to address this hypothesis using a large set of published PBMC repertoires (Figure 3A).

We determined the proportion of repertoires from a cohort of 786 individuals collected and sequenced pre-SARS-Cov-2 (referred to here as the Emerson dataset),^28^ which contain each of the expanded CDR3 sequences (associated with the same V gene) as those defined in our study. Since each repertoire from the Emerson set contains only a small sample of the total repertoire in each individual (on average 1.8∗10^5^ sequences per sample), the inter-individual sharing must reflect a high abundancy of the shared TCR in the sample. The average abundance of the TCR in the repertoire can be estimated for all those TCRs which are seen at least twice in the Emerson dataset using the Poisson probability distribution. Specifically, the proportion of the individuals which do NOT contain a particular sequence (p(X = 0)) is given by

(Equation 1) $$p\;\left(X=0\right)=e^{\left(-\mu \right)}$$

where μ is the average number of times a TCR is observed in a sample. The average number of TCRs sequenced per sample in the Emerson data is 1.8∗10^5^. Therefore the frequency of the TCR in the repertoire, in TCRs/million can be estimated by

(Equation 2) $$f=\frac{-\log\left(p\left(X=0\right)\right)*10^{6}}{1.8^{*}10^{5}}$$

**Figure 4:**
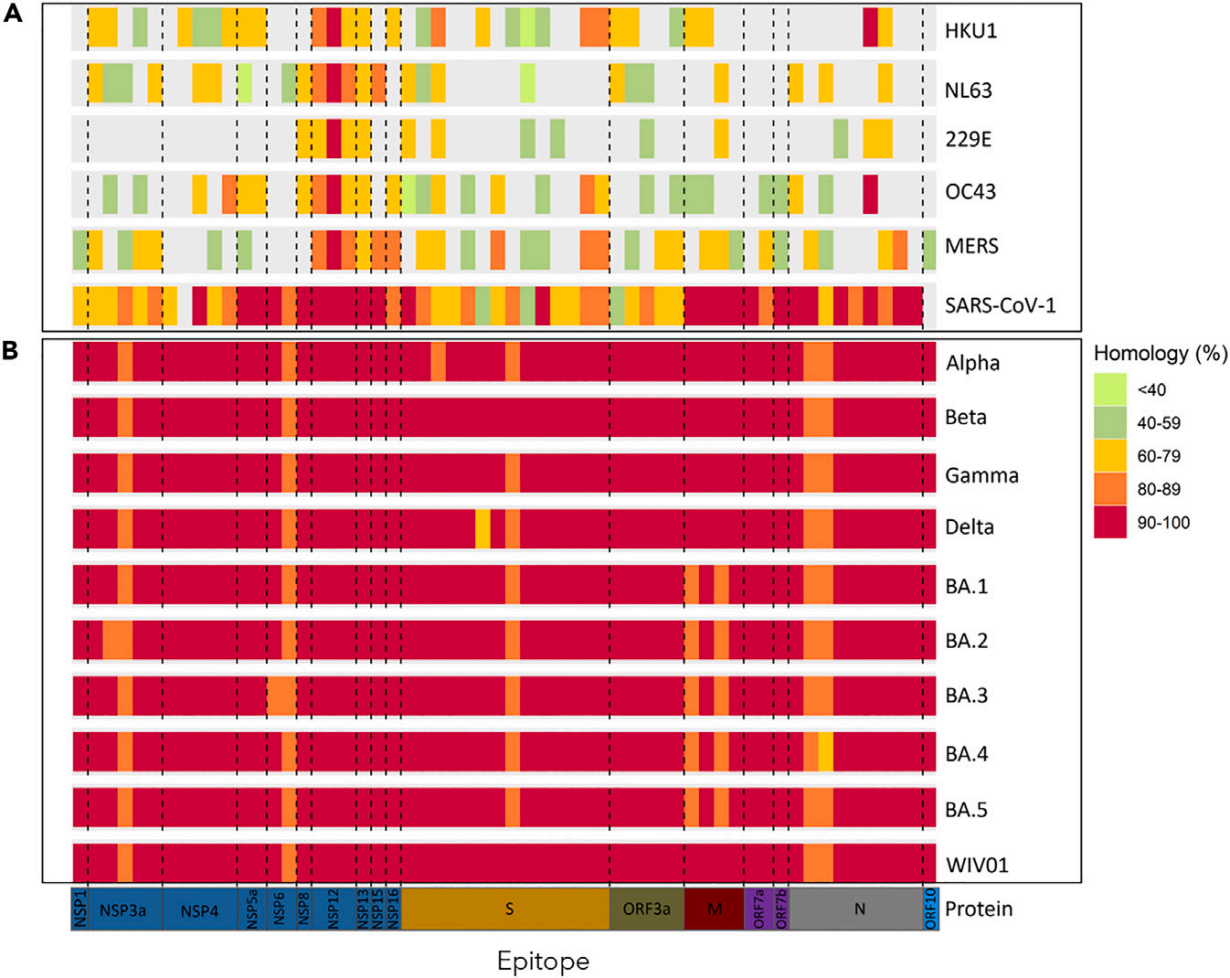
Expanded TCRs in infected hosts cannot be explained by cross-reactivity to other coronaviruses (A) Circulating coronaviruses. (B) Different strains of SARS-COV-2. Each column is a functionally defined T cell epitope of SARS-COV-2 which is recognized by a TCR whose sequence is found among the set of TCRs expanding following infection with the virus (as defined in Figure 1). The degree of homology is shown by color coding.

As shown in Figure 3B the proportion of these SARS-COV-2 naive individuals in whom we found each of the SARS-COV-2 expanded TCR is much greater than for non-expanded TCRs from the same repertoires. 2648 (52%) of the 5139 unique TCR beta expanded sequences (the Emerson data is restricted to beta sequences) were observed two or more times in the Emerson dataset. The estimated frequency distribution of these expanded TCRs estimated using Equation 2 is plotted in Figure 3C, and compared to non-expanded TCRs. The mean frequency of the expanded set is 1.8 per million, over a thousand times higher than 1 per 10^9^, which as discussed above is the estimated frequency of individual TCR sequences in the naive repertoire. We further investigated whether the expanding TCRs could be sub-divided further based on whether they peaked at week 0/1 or week 4 (see Figure S13A). However, there was no clear cut distinction between these two sets of TCRs (Figures 3B and 4C), perhaps because of the small number of TCRs in the 4 weeks set. We note that we did not match CDR3s in our data and those in Emerson for HLA type, since we did not know the correct HLA restriction of each TCR, which adds further noise to the analysis. Individual CDR3s can be characterized by their probability of generation^29^ by somatic recombination. We calculated the probability of generation of each expanded TCR using the OLGA algorithm.^30^ As predicted by their increased frequency in the Emerson data, the expanded TCRs had higher probabilities of generation than a random set of TCRs (Figure S12).

### Expanded TCRs in infected hosts cannot be explained by cross-reactivity to other coronaviruses

We have previously demonstrated that human TCR repertoire has a very broad range of TCR frequencies.^31^ High-frequency precursor T cells could therefore arise either because they arise from naive T cells with a higher than average frequency or due to cross-reaction.^9^^,^^32^ The samples were collected during the first wave of SARS-COV-2 in the UK, and the response could not therefore be attributed to pre-existing memory responses against SARS-COV-2. We therefore explored the hypothesis that the early wave of TCRs represented a restimulation of cross-reactive memory cells present as a result of exposure to common circulating human coronaviruses (hCoVs). In order to test this hypothesis, we analyzed the 65 peptide epitopes which were recognized by the 141 TCRs which were functionally annotated as SARS-COV-2 specific and were also identified as early expanders in our dataset (as detailed in Figure 2). The similarity between these peptide epitopes and the homologous sequences from the major strains of circulating hCoVs is shown in Figure 4A. In general, with the exception of one NSP12 (polymerase) epitope, the SARS-COV-2 sequences in the hCoVs showed substantial differences from their homologous sequences in SARS-COV-2, making it unlikely that the TCRs specific for these sequences would recognize the equivalent peptide from the circulating hCoVs. We cannot, however, exclude the possibility of cross-reactivity with some other unrelated and unidentified pathogen.^9^^,^^32^ In contrast, the epitopes recognized by these early annotated TCRs showed extremely high homology between the different variants of SARS-COV-2 (Figure 4B). Within spike protein, for example, the early T cell epitopes were distinct from the mutations which are known to affect antibody recognition (Figure S13).^33^ This analysis does not in any way preclude the existence of T cells which cross-react between SARS-COV-2 and circulating hCoVs as suggested in other studies.^9^^,^^34^ Rather, the analysis argues that the observed rapid expansion of SARS-COV-2-specific TCRs can occur even in the absence of any such cross-reaction.

### High-frequency LCMV specific TCRs in the naive repertoire

**Figure 5:**
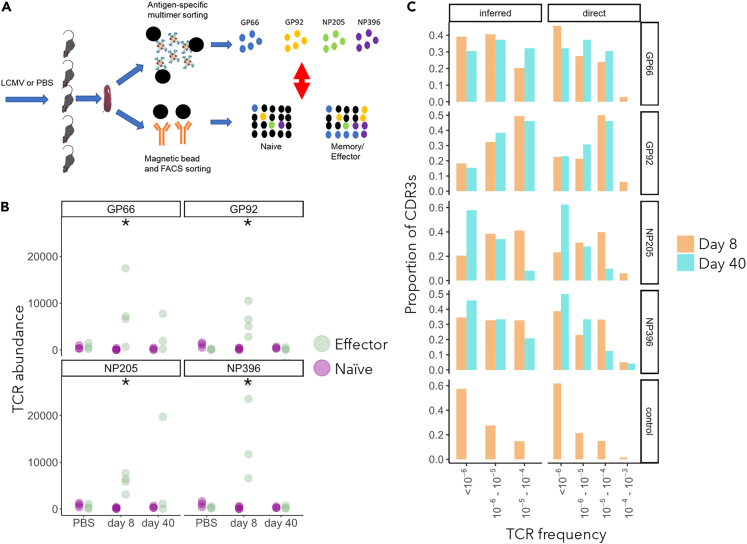
High-frequency LCMV specific TCRs in the naïve repertoire (A) A schematic of the experimental design. Mice were injected with PBS or LCMV and spleen T cells harvested at day 8 and day 40 post-infection. Epitope-specific T cells were isolated using specific tetramer sorting, and different subpopulations of bulk T cells were fractionated by a combination of FACS sorting and magnetic bead/antibody sorting. Both sets of T cells were processed for TCR sequencing and the overlap between the populations analyzed. (B) Total abundance of epitope-specific TCRs in the naive (purple) or effector (green) TCR repertoires in individual mice. The abundance of all four epitopes was larger in the effector population in the day 8 infected group, than in the PBS group (∗p < 0.001, Mann Whitney test). All other pairwise comparisons were not significant. (C) Distribution histogram of frequency of epitope-specific and control TCRs, estimated using either the statistical Poisson framework as in Figure 3 (left panels), or directly from TCR abundance data (right panels). Epitope-specific CDRs are split into CDRs that come up early (day 8, orange) and CDRs that come up later (day 40, cyan). The left column includes all epitope TCR which were not found within the bulk naive population, and were assigned a frequency of less than 10^−6^.

The number of viable cells collected at early timepoints of the COVIDSortium study limited the types of analysis we could carry out, and hence the ability to fully investigate the alternative hypothesis that the expanding TCRs we identified were derived from large pre-existing T cell clones in the naive repertoire. However, we reasoned that the phenomenon we observed for SARS-COV-2 might be common to other viral infections, and might reflect fundamental features of the naive T cell repertoire. We therefore examined the abundances of TCRs from C57BL/6 mice exposed to LCMV. This model has been studied extensively, and several dominant and sub-dominant epitopes have been described.^35^^,^^36^^,^^37^ We harvested spleens from mice at day 8 and day 40 post-infection with saline or LCMV. In contrast to the SARS-COV-2 data, we did not have to rely on dynamics to identify virus specific T cells. Instead we directly isolated antigen-specific T cells by sorting with fluorescent MHC tetramers bound to four well-described LCMV epitopes: GP66, GP92, NP205, and NP396. An aliquot of spleen cells from each animal was separately fractionated into naive (CD62L+, CD44^−^), central memory (CD62^+^, CD44^−^) and effector (CD62^−^, CD44^+^) phenotype as described in STAR Methods. We sequenced the TCR repertoire of each antigen-specific and non-specific sample, and then searched for antigen-specific TCR (tetramer-bound) sequences in the repertoire of the bulk naive and effector populations (Figure 5A).

There was a strong enhancement in the abundance of epitope-specific TCRs in the effector compartment of LCMV infected mice, especially at day 8 postinfection, compared to saline injected mice (Figure 5B). In contrast, although many epitope-specific TCRs were detected in the naive repertories, their abundance did not change between infected and PBS treated mice. Thus detection of epitope-specific TCRs in the naive repertories is unlikely to arise from contamination during the fractionation step.

We then examined the frequency distribution of the epitope-specific TCRs in the naive repertoires (Figure 5C). To evaluate the difference between TCRs that expand early (which might have higher precursor frequency) and TCRs which expand later, we classified the TCRs as day 8 (already detected in the bulk sequencing at day 8) or day 40 (not yet detected in bulk sequencing at day 8, but detected at day 40, Figure S14A). As shown in Figure 5C, in 3 out of 4 epitopes the early rising antigen-specific TCRs were enriched for high-abundance CDR3s, compared to the antigen-specific CDRs which arose at a later time point, or to a random sample of TCRs from the non-antigen-specific repertoires (compare top four rows to bottom row). Because we used a quantitative TCR sequencing protocol, we were able to estimate frequency by two orthogonal methods. In the left column, we show the frequencies estimated using the Poisson sampling approach which we developed for the SARS-COV-2 data above. In brief, we determined the proportion of naive repertoires in which we detected each TCR, and then used Equation 2 to infer its frequency in the repertoire. Because we only had data from 10 mice, the granularity of this approach in this case was limited. In a second approach (right panels), we measured the average frequency of each TCR directly (counts/total number of TCR in sample), and then averaged the frequencies overall 10 mice. TCRs not detected were assigned a frequency less than 10^−6^ in both methods. Both methods of frequency estimation gave very similar results (Figure S14B), providing additional confidence in the statistical approach used for SARS-COV-2. The early (day 8) TCRs also showed a higher generation probability distribution^30^ than the late TCRs, consistent with their detection more often in naive repertoires (Figure S14C). Overall, these results suggest that high-frequency TCRs specific for LCMV exist in the naive repertoire prior to LCMV exposure.

## Discussion

The high prevalence and high degree of synchrony of the first wave of the SARS-COV-2 pandemic in the UK offered a rare opportunity to study the early dynamics of the T cell receptor repertoire following exposure to a natural novel viral infection. We identified a small number of TCRs which showed statistically significant changes in abundance within the first five weeks of our study, reasoning that viral exposure would lead to T cell clonal expansion and subsequent contraction. The set of TCRs identified in this way created a rapid transient wave of expansion, which peaked at or shortly after the first PCR+ SARS-COV-2 test. The number of expanded TCRs varied between individuals within a range of 18–912, and constituted only a very small fraction (1–2%) of the repertoire, consistent with the small proportion of activated T cells observed in blood using epitope-specific tetramers or peptide specific T cell cytokine production.^38^^,^^39^

The identification of the responding TCRs was unbiased and based on dynamics alone. The wave of expansion was not observed in PCR- controls, consistent with a SARS-COV-2 specific response. However, the rapid expansion of TCRs might arise from cytokine-driven antigen non-specific as well as antigen-specific clonal expansion. Access to a number of large sets of CDR3s annotated as SARS-COV-2 specific, as well as similar sets annotated for CMV and EBV specificity by functional assays allowed us to interrogate the expanding set for antigen specificity. The expanded set of CDR3s were enriched for SARS-COV-2 specific annotated TCRs, although we cannot rule out that some bystander activation of other memory cells may occur. The annotated CDR3s observed in our dataset shared V gene usage with the V gene of the corresponding annotated TCRs. Furthermore, most expanded annotated TCRs were found in individuals expressing the same HLA allele as the reported restriction element of the annotated TCR. Both these observations build confidence in the functional annotation of the expanded TCRs as specific for SARS-COV-2.

The very early detection of highly expanded T cell clonotypes suggests the T cell precursors were present at high frequencies even before exposure to SARS-COV-2. In support of this hypothesis we were able to detect many of the expanded CDR3 sequences of the expanded TCRs in a significant proportion of pre-pandemic repertoires. We reasoned that TCRs which are present in many individuals (the public repertoire) must be present at high frequency in order to be observed in the small sample of blood which is typically sequenced. For example, in the Emerson dataset of 786 repertoires collected and sequenced several years before the SARS-COV-2 pandemic, an average 100,000 TCR sequences are sequenced for each individual, out of an estimated total number of 10^11^ -10^12^.^40^ We were therefore able to estimate the underlying frequency distribution of the 2648 expanded CDR3 TCRbeta sequences which we observed in two or more of the Emerson repertoires. As we predicted, the observed frequency distribution of these TCRs was between 10^−7^ and 10^−5^, with an average frequency of 1.8 ∗ 10^−6^. At least 50% of the TCRs which expanded therefore had high precursor frequency in pre-COVID repertoires of healthy individuals. Rapid T cell responses to live viral vaccines, which act as a good model of acute viral infection, have been reported previously^41^^,^^42^ and similar analysis of the TCR sequences expanded in these studies may further extend the generality of this phenomenon.

We considered two possible hypotheses to explain the presence of high precursor frequency SARS-COV-2 specific T cells in the pre-pandemic repertoire. The first hypothesis is that these TCRs are present on cross-reactive memory T cells specific for homologous epitopes on circulating human coronaviruses. Similar cross-reactive T cells, especially directed at the conserved polymerase, have recently been described and may explain “abortive” infections with SARS-COV-2.^9^ However, an analysis of the epitopes recognized by the annotated early expanding TCRs identified in this study (which are predominantly directed at structural proteins) showed little conservation between SARS-COV-2 and the main circulating human coronaviruses (Figure 4). This suggests that the early responding T cells we document in this study do not arise from the main circulating human coronaviruses. We cannot, however, rule out the possibility that these T cells arise from pre-existing memory T cells cross-reactive for other unknown pathogens. The epitopes recognized by the early expanding T cells were highly conserved (in most cases identical) between all different strains of SARS-COV-2. There is no evidence, therefore, that evolution has driven escape mutations within this set of T cell epitopes, similar to that seen for antibody. An important limitation of our study, however, is that the range of disease severity was very limited, and largely restricted to mild or asymptomatic disease. The potential role of early expanding T cells in limiting viral growth and hence pathology^43^ cannot be investigated in this study, since all the infected HCW only developed mild disease.

The second hypothesis we explore is that the early SARS-COV-2-specific T cell response we observe can be attributed to TCRs present at high precursor frequency in the naive compartment. Recent estimates suggest that the naive repertoire may comprise 10^8^- 10^9^ different TCRs,^44^ but the frequency distribution is very broad with some TCRs present as high as 1 in 10^4^.^31^ Several factors may drive differential frequency of different T cell receptors in the circulation, although the mechanisms remain incompletely understood. We could not measure the frequency of individual TCRs in the pre-infection naive repertoires of the HCW from this cohort since we did not have sufficient stored PBMC to sort and sequence the naive compartment prior to infection.

We reasoned that the phenomenon of rapidly responding high-frequency T cells in the naive repertoire might be a more general feature of the T cell adaptive immune system. We therefore investigated one of the classical most well studied models of virus infection, LCMV.^45^ We isolated epitope-specific T cells expanding very early after infection (day 8) and searched for TCRs with identical CDR3s in the naive, as well as effector repertoires of both immunized and unimmunized mice. The results we obtained were very similar to those we had observed with SARS-COV-2, but in this case we were able to directly test our principal hypothesis that T cells with high endogenous frequency are present within the naive compartment, and contribute significantly to the early wave of T cell responses following infection. Moreover, we could detect a difference in the distribution of frequency in the naive repertoire between cells that arise early and cells that are detected later in the infection, further supporting our hypothesis that early expanding cells have higher precursor frequencies in the naive compartment.

Our study raises the intriguing possibility that the human immune system contains a population of high-frequency precursor T cells which can provide a very rapid response to viral infections. These rapid responses do not require cross-reaction with circulating human coronaviruses, but are associated with pre-existing high-frequency precursors. Further studies will be required to determine whether these are high-frequency naive T cells, or represent random cross-reactivity of the memory pool. The ability to stimulate this rapid response may be an important factor in designing future vaccines to limit the growth and hence pathogenesis of respiratory viral infections.

### Limitations of the study

The design of the COVIDsortium study imposed some limitations. The protocol of the study, which was set up as rapidly as possible to capture the first wave of the SARS-COV-2 pandemic in the UK, did not incorporate collection of sufficient number of viable cells at the early time points to carry out functional antigen-specific assays. Although we did not observe a wave of early TCR expansion/contraction in PCR-seronegative individuals, we cannot therefore exclude that some of the expanding TCRs were bystander or cross-reactive memory cells to other antigens. Furthermore, as discussed above, the same limitations on numbers of stored cells did not allow us to test directly our principal hypothesis that the early expanding TCRs arose from high frequency pre-exposure naive T cell clones.

A second limitation was that the HLA restriction of most of the expanding and contracting TCRs is unknown. The comparison with the pre-pandemic repertoires is therefore carried out without reference to HLA, which may limit the interpretation.

Finally the range of disease severity in this cohort was very limited, and largely restricted to mild or asymptomatic disease. The potential role of early expanding T cells in limiting viral growth, and hence pathology cannot be investigated in this study, since all the infected HCW only developed mild disease.

## STAR★Methods

### Key resources table

**Table:** 

REAGENT or RESOURCE	SOURCE	IDENTIFIER
**Antibodies**		

Percp/cy5.5 CD4 mouse	Biolegend	Cat# 100433; RRID: AB_893330
Percp/cy5.5 CD8 mouse	Biolegend	Cat# 100733;RRID: AB_2075239
PE CD3 mouse	Biolegend	Cat# 100205;RRID: AB_312662
APC CD62L mouse	Biolegend	Cat# 104411;RRID: AB_313098
FITC CD44 mouse	Biolegend	Cat# 103005;RRID: AB_312956

**Bacterial and virus strains**		

LCMV Armstrong		

**Chemicals, peptides, and recombinant proteins**		

H-2D(b) LCMV NP 396-404 FQPQNGQFI	NIH Tetramer Core Facility	
H-2K(b) LCMV NP 205-212 YTVKYPNL	NIH Tetramer Core Facility	
H-2D(b) LCMV GP92-101 CSANNSHHYI	NIH Tetramer Core Facility	
H-2A(b) LCMV GP66–77 DIYKGVYQFKSV	NIH Tetramer Core Facility	

**Critical commercial assays**		

Pan T Cell Isolation Kit II, mouse	Miltenyi Biotec	Cat #130-095-130
CD3ε MicroBead Kit, mouse	Miltenyi Biotec	Cat #: 130-094-973
CD4^+^ T Cell Isolation Kit, mouse	Miltenyi Biotec	Cat #: 130-104-454
CD8a+ T Cell Isolation Kit, mouse	Miltenyi Biotec	Cat #: 130-104-07
SMARTer TCR a/b Profiling Kit, human	Takara	Cat. # 634478

**Deposited data**		

COVIDsortium TCR sequencing data	This paper	SRA:PRJNA718557
LCMV mouse model TCR sequencing data	This paper	SRA:PRJNA771880, PRJNA954849
Emerson et al.^28^ pre-pandemic TCR beta sequences from healthy volunteer	immuneACCESS	https://doi.org/10.21417/B7001Z

**Experimental models: Organisms/strains**		

Mouse: 5 weeks old (Envigo) C57BL6 female	Jackson Laboratories	Strain #:**000664**

**Software and algorithms**		

Decombinator software v4.2	Oakes et al. 2017^46^; Uddin et al., 2019^47^; Peacock et at, 2021^48^	https://github.com/innate2adaptive/Decombinator
R v4.2.2	R Core Team, 2021^49^	https://www.R-project.org/
BLASTp v2.11.0	Sawyers et al., 2022^50^	https://blast.ncbi.nlm.nih.gov/Blast.cgi?PAGE=Proteins

**Other**		

Custom scripts to reproduce analyses described	This paper	https://github.com/innate2adaptive/CovidsortiumTCRExpanded/
Custom scripts to perform the homology mapping of SARS-CoV-2 epitopes	This paper	https://github.com/cednotsed/early_tcell_x-reactivity.git
Clinical Trial - COVIDsortium	NCT04318314	ClinicalTrials.gov

### Resource availability

#### Lead contact

Further information and requests for resources and reagents should be directed to and will be fulfilled by the lead contact, Prof Benny Chain (b.chain@ucl.ac.uk).

#### Materials availability

This study did not generate new unique reagents.

### Experimental model and study participant details

#### COVIDsortium healthcare study cohort

Healthcare workers were recruited at St Bartholomew’s Hospital, London, UK in the week of lockdown in the United Kingdom (between 23rd and 31st March 2020).^3^ Participants underwent weekly evaluation using a questionnaire and biological sample collection (including serological assays) for up to 16 weeks when fit to attend work at each visit, with further follow up samples collected at 6 months. Participants with available blood RNA samples who had PCR-confirmed SARS-CoV-2 infection (Roche cobas diagnostic test platform) at any time point were included as ‘cases’. A subset of consecutively recruited participants without evidence of SARS-CoV-2 infection on nasopharyngeal swabs and who remained seronegative by both Euroimmun antiS1 spike protein and Roche anti-nucleocapsid protein throughout follow-up were included as uninfected controls.

#### Ethical approval

The COVIDsortium study was approved by a UK Research Ethics Committee (South Central - Oxford A Research Ethics Committee, reference 20/SC/0149). All participants provided written informed consent. The animal studies were performed according to regulations formulated by The Weizmann Institute’s Animal Care and Use Committee.

#### COVIDsortium study design

We undertook a case control study nested within our COVIDsortium health care worker cohort. Participant screening, study design, sample collection, and sample processing have been described in detail previously^3^^,^^39^ and the study is registered at ClinicalTrials.gov (NCT04318314). Briefly, healthcare workers were recruited at St Bartholomew’s Hospital, London, UK in the first week of lockdown in the United Kingdom (between 23^rd^ and 31^st^ March 2020). In London, the case-doubling time in March, 2020 was approximately 3–4 days. The number of nasal swabs testing positive for SARS-CoV-2 in our study peaked at March 23rd to 31st, 2020 suggesting that infections peaked on or around March 23rd, 2020, the day of UK lockdown. We thus observed approximately synchronous infections coincident with the peak epidemic transmission in London at the start of the study, UK lockdown on March 23rd and therefore used this as the benchmark starting point for our analysis in the first wave. Participants underwent weekly evaluation using a questionnaire and biological sample collection (including serological assays) for up to 16 weeks when fit to attend work at each visit, with further follow up samples collected at 6 months.

Participants with available blood RNA samples who had PCR-confirmed SARS-CoV-2 infection (Roche cobas® diagnostic test platform) at any time point were included as ‘cases’. Six participants without evidence of SARS-CoV-2 infection on nasopharyngeal swabs and who remained seronegative by both Euroimmun anti-S1 spike protein and Roche anti-nucleocapsid protein throughout follow-up were included as uninfected controls.

Further demographic details of the cohort are found in Figure S2.

#### LCMV mouse models

##### LCMV infections

Seven 5 weeks old (Envigo) C57BL/6 female mice were injected intraperitoneally with 2 x 10^5^ units plaque forming units of the Armstrong LCMV strain. Mice were sacrificed after 8 or 40 days of infection, and spleens were collected. An additional 3 mice were injected with saline and sacrificed on day 8. All mice were maintained in specific pathogen-free conditions.

### Method details

#### Genotyping

Samples were genotyped using Illumina Infinium Global Screening Array-24v1+MD.^51^ Quality control and filtering was carried out in PLINK v1.90b6.12^52^ For each sample HLA alleles A, B, C, DQA1, DQB1, DPB1 and DRB1 were imputed using the HLA Genotype Imputation with Attribute Bagging (HIBAG) v1.24.0 package in R v4.0.1.^53^ The publicly available HLARES and HapMap Phase 2 datasets, genotyped using the same array as the input data, were used as reference for the imputation. Imputation accuracy was increased by inclusion of ethnicity-specific reference panels.

#### T cell receptor sequencing

The α and β chains of the TCR repertoire were sequenced from all time points for which RNA was available within the first 4 weeks of the study for all participants who were PCR+ at any time point, and for six randomly selected individuals who remained PCR and seronegative throughout the study. The pipeline introduces unique molecular identifiers attached to individual cDNA molecules using single-stranded DNA ligation The UMI allows correction for sequencing error PCR bias, and provides a quantitative and reproducible method of library preparation.^46^^,^^47^^,^^48^

T cell cloning and single cell TCR sequencingTCR sequences of SARS-CoV-2 specific T cells were obtained as previously described.^23^ In brief, antigen-specific CD4^+^ and CD8+T cells were stained with peptide-MHC class I pentamer and peptide-MHC class II tetramer, respectively, and were then sorted by flow cytometry for single cell RNASeq. T cell receptor sequences of each single cell were reconstructed from Smartseq2 scRNA-seq FASTQ files using MiXCR v.3.0.13,^54^ or extracted from 10x VDJ sequencing using cellranger vdj (Cellranger). Deep sequencing of TCR repertories of T cell clones was carried out using a SMARTer human TCR a/b profiling Kit (Takara) following the supplier’s instructions..

In order to assess the sensitivity of the SARS-CoV-2 specific T cells, SARS-CoV-2 specific T cell clones were generated by limiting dilution, as previously described.^23^ T cell clones were then co-cultured with target cells loaded with peptide at titrated concentration. Cytokine production of each T cell clone was then assessed by IFN-gamma ELISpot or intracellular cytokine staining. EC50 of the T cells were then calculated by Prism using nonlinear regression with variable slope. T cell clones with lower EC50 are more sensitive to antigen stimulation.

#### Mouse T cell isolation

Spleens were dissociated with a syringe plunger and single cell suspensions treated with ammonium-chloride potassium lysis buffer to remove erythrocytes. Bone marrows were extracted from the femur and tibiae of the mice and washed with PBS. Samples were loaded on MACS column (Miltenyi Biotec) and T cells were isolated according to manufacturer’s protocol. Bone marrows cells were purified with CD3^+^ T isolated kit (CD3ε Micro-Bead Kit, mouse, 130-094-973, Miltenyi Biotec). Splenic CD4^+^ and CD8^+^ cells were purified in two steps: (1) CD4^+^ positive selection (CD4^+^ T Cell Isolation Kit, mouse, 130-104-454, Miltenyi) (2) the negative cell fractions were further selected for the CD8^+^ positive cells (CD8a+ T Cell Isolation Kit, mouse, 130-104-07, Miltenyi Biotec). For the tetramer binding reaction, we pooled splenocytes from previously infected mice (5 mice after 8 days of infection) and purified their T cells using the T cell isolation kit (Pan T Cell Isolation Kit II, mouse, 130-095-130, Miltenyi Biotec).

#### Mouse flow cytometry analysis and cell sorting

The following fluorochrome-labeled mouse antibodies were used according to the manufacturers’ protocols: Percp/cy5.5 CD4 100433, PerCP/cy5.5 CD8, PE CD3, APC CD62L, FITC or PE/cy7 anti- CD44 (Biolegend). Cells were sorted on a SORP-FACS-AriaII and analyzed using FACSDiva (BD Biosciences) and FlowJo (Tree Star) software.^55^ Sorted cells were centrifuged (450g for 10 minutes) prior to RNA extraction.

#### LCMV-tetramer staining and FACS sorting

Four monomers (NIH Tetramer Core Facility) with different LCMV epitopes were used: MHCI- NP396-404(H-2Db), MHCI- NP205-212(H-2Kb), MHCI- GP92-101 (H-2Db) and MHCII-GP66–77(H-2Ab). Tetramers were constructed by binding biotinylated monomers with PE/APC – conjugated- streptavidin (according to the NIH protocol). Purified T cells were stained with FITC anti-CD4 and PE anti-CD8 followed by tetramer staining (two tetramers together), for 30 min at room temperature (0.6ug/ml). CD8^+^ or CD8^+^ epitope-specific cells were sorted from single-positive gates for one type of tetramer (Figure S15).

### Quantification and statistical analysis

All statistical analysis in the paper was carried out in R v4.2.2.^56^ Where relevant, exact details of statistical tests are provided in the figure legend.

#### Identification of expanded TCRs

Expanded TCRs were defined as any TCR which changed significantly between any two time points. The significance boundaries (shown as blue dotted lines in Figure 1A) were defined as the maximum TCR abundance which might be observed at time 2, given its abundance at time 1, given Poisson distribution of counts with p < 0.0001, to give a False Discovery Rate of <1 in 1000. TCR abundances are normalized for total number of TCRs sequenced in each sample, and expressed as counts/million. MAIT TCRs were defined as any TCR alpha containing TRAV1-2 paired with TRAJ12, TRAJ20 or TRAJ33. iNKT TCRs were defined as TCRs containing TRAV10 paired with TRAJ18.

#### TCR clustering

The pairwise distance between expanded or control CDR3 sequences was measured by measuring the number of shared triplet amino acid motifs between the two CDR3s, and normalizing by the length of the CDR3 to give a similarity metric between 1 (identity) and zero (no sharing). The analysis was done using the stringdot and kernelMatrix functions in the kernlab package V0.9-31. The similarity matrix was plotted as a graph (layout format fruchterman-reingold), using the package igraph version 1.2.8^57^ implemented in R. Each CDR3 is represented as a node, and nodes are connected by an edge if the triplet similarity is greater than a given threshold. The thresholds used for clustering were 0.76 for CDR3α and 0.72 for CDR3β, to give a FDR in similar size control sets of 1 in 10,000.

#### Emerson data set

The Emerson dataset was downloaded from the link provided in.^28^ We used all 786 samples (cohort and control), and computed for each CDR3 its sharing level in the cohort. The sharing level of an amino acid CDR3 sequence is defined by the number of repertoires in the cohort that contain the given CDR3.

#### Homology mapping of SARS-COV-2 epitopes

We used blastp from BLAST+ v2.11.0^50^ to compute the sequence homology of the 59 unique epitope sequences against a database of protein sequences derived from all seven species of existing human-associated coronaviruses (HCoVs). We used the same parameters as our previous study, which are optimized for short query sequences.^58^ Briefly, -task, -qcov_hsp_perc, -num_alignments, -evalue were set to blastp-short, 99, 109 and 2 × 109, respectively.

To construct the blastp database, we retrieved all genome records for HCoV-NL63 (taxid:277944; n = 71), HCoV-229E (taxid:11137; n = 41), HCoV-HKU1 (taxid:290028; n = 33) and HCoV-OC43 (taxid:31631; n = 206) on NCBI Virus. Only accessions for genomes isolated from human hosts and flagged as ‘complete’ were retained. Protein sequences for all accessions were downloaded using the online Batch Entrez utility (https://www.ncbi.nlm.nih.gov/sites/batchentrez). Separately, we retrieved the WIV01 reference and a random sample of 50 genomes for each existing variant of concern (VoC): Alpha, Beta, Gamma, Delta, and Omicron from the 15th June Audacity release on GISAID.^59^^,^^60^ Protein annotations of WIV01 were obtained from GenBank (accession: NC_045512.2) and used to obtain protein sequence annotations from the SARS-CoV-2 genomes. To do so, we aligned the SARS-CoV-2 genomes against WIV01 using the Augur v14.0.04 wrapper for MAFFT v7.490.^61^^,^^62^ Protein sequences were translated from the aligned SARS-CoV-2 genomes using the trans function as part of the Ape v5.6.2 package in R.^63^ All public databases were accessed on 20th June 2022.

### Additional resources

Full COVIDsortium details: https://clinicaltrials.gov/ct2/show/NCT04318314?term=NCT04318314&draw=2&rank=1

The Decombinator software used for analysis of TCRsequencing data is freely available at: https://github.com/innate2adaptive/Decombinator.

All scripts needed to reproduce the analysis in the paper are freely available at: https://github.com/innate2adaptive/CovidsortiumTCRExpanded.

## Data Availability

• The COVIDsortium TCR sequencing data have been deposited at NCBI Sequence Read Archive (https://www.ncbi.nlm.nih.gov/sra) and mouse TCR sequencing data have been deposited at Sequence Read Archive) and are publicly available under project accession number SRA:PRJNA718557. All other data are available in the main text or the supplemental information. • All code for analysis and generating individual figure panels are available on GitHub, and links to the repositories are provided in the key resources table. • Any additional information required to reanalyse the data reported in this paper is available from the lead contact upon request. Requests will be responded to within 4 weeks of receipt.

## References

[1] Co M.D.T., Kilpatrick E.D., Rothman A.L. (2009). Dynamics of the CD8 T-cell response following yellow fever virus 17D immunization. Immunology.

[2] Althaus C.L., Ganusov V.v., de Boer R.J. (2007). Dynamics of CD8 + T cell responses during acute and chronic lymphocytic choriomeningitis virus infection. J. Immunol..

[3] Treibel T.A., Manisty C., Burton M., McKnight Á., Lambourne J., Augusto J.B., Couto-Parada X., Cutino-Moguel T., Noursadeghi M., Moon J.C. (2020). COVID-19: PCR screening of asymptomatic health-care workers at London hospital. Lancet.

[4] Moss P. (2022). The T cell immune response against SARS-CoV-2. Nat. Immunol..

[5] Grifoni A., Sidney J., Vita R., Peters B., Crotty S., Weiskopf D., Sette A. (2021). SARS-CoV-2 human T cell epitopes: adaptive immune response against COVID-19. Cell Host Microbe.

[6] Tarke A., Sidney J., Kidd C.K., Dan J.M., Ramirez S.I., Yu E.D., Mateus J., da Silva Antunes R., Moore E., Rubiro P. (2021). Comprehensive analysis of T cell immunodominance and immunoprevalence of SARS-CoV-2 epitopes in COVID-19 cases. Cell Rep. Med..

[7] Stephenson E., Reynolds G., Botting R.A., Calero-Nieto F.J., Morgan M.D., Tuong Z.K., Bach K., Sungnak W., Worlock K.B., Yoshida M. (2021). Single-cell multi-omics analysis of the immune response in COVID-19. Nat. Med..

[8] Yoshida M., Worlock K.B., Huang N., Lindeboom R.G.H., Butler C.R., Kumasaka N., Dominguez Conde C., Mamanova L., Bolt L., Richardson L. (2022). Local and systemic responses to SARS-CoV-2 infection in children and adults. Nature.

[9] Swadling L., Diniz M.O., Schmidt N.M., Amin O.E., Chandran A., Shaw E., Pade C., Gibbons J.M., le Bert N., Tan A.T. (2022). Pre-existing polymerase-specific T cells expand in abortive seronegative SARS-CoV-2. Nature.

[10] Kedzierska K., Thomas P.G. (2022). Count on us: T cells in SARS-CoV-2 infection and vaccination. Cell Rep. Med..

[11] Goldblatt D., Alter G., Crotty S., Plotkin S.A. (2022). Correlates of protection against SARS-CoV-2 infection and COVID-19 disease. Immunol. Rev..

[12] Heitmann J.S., Bilich T., Tandler C., Nelde A., Maringer Y., Marconato M., Reusch J., Jäger S., Denk M., Richter M. (2022). A COVID-19 peptide vaccine for the induction of SARS-CoV-2 T cell immunity. Nature.

[13] Minervina A.A., Komech E.A., Titov A., Bensouda Koraichi M., Rosati E., Mamedov I.Z., Franke A., Efimov G.A., Chudakov D.M., Mora T. (2021). Longitudinal high-throughput tcr repertoire profiling reveals the dynamics of t-cell memory formation after mild covid-19 infection. Elife.

[14] Wilkinson T.M., Li C.K.F., Chui C.S.C., Huang A.K.Y., Perkins M., Liebner J.C., Lambkin-Williams R., Gilbert A., Oxford J., Nicholas B. (2012). Preexisting influenza-specific CD4+ T cells correlate with disease protection against influenza challenge in humans. Nat. Med..

[15] Chandran A., Rosenheim J., Nageswaran G., Swadling L., Pollara G., Gupta R.K., Burton A.R., Guerra-Assunção J.A., Woolston A., Ronel T. (2022). Rapid synchronous type 1 IFN and virus-specific T cell responses characterize first wave non-severe SARS-CoV-2 infections. Cell Rep. Med..

[16] Bernal Lopez J., Panagiotopoulos N., Byers C., Vilaplana T.G., Boddington N., Zhang X.S., Charlett A., Elgohari S., Coughlan L., Whillock R. (2022). Transmission dynamics of COVID-19 in household and community settings in the United Kingdom, January to March 2020. Euro Surveill..

[17] Lauer S.A., Grantz K.H., Bi Q., Jones F.K., Zheng Q., Meredith H.R., Azman A.S., Reich N.G., Lessler J. (2020). The incubation period of coronavirus disease 2019 (CoVID-19) from publicly reported confirmed cases: estimation and application. Ann. Intern. Med..

[18] Backer J.A., Klinkenberg D., Wallinga J. (2020). Incubation period of 2019 novel coronavirus (2019- nCoV) infections among travellers from wuhan, china, 20 28 january 2020. Euro Surveill..

[19] McAloon C., Collins Á., Hunt K., Barber A., Byrne A.W., Butler F., Casey M., Griffin J., Lane E., McEvoy D. (2020). Incubation period of COVID-19: a rapid systematic review and meta-analysis of observational research. BMJ Open.

[20] Manisty C., Treibel T.A., Jensen M., Semper A., Joy G., Gupta R.K., Cutino-Moguel T., Andiapen M., Jones J., Taylor S. (2021). Time series analysis and mechanistic modelling of heterogeneity and sero-reversion in antibody responses to mild SARS-CoV-2 infection. EBioMedicine.

[21] Shugay M., Bagaev D.V., Zvyagin I.V., Vroomans R.M., Crawford J.C., Dolton G., Komech E.A., Sycheva A.L., Koneva A.E., Egorov E.S. (2018). VDJdb: a curated database of T-cell receptor sequences with known antigen specificity. Nucleic Acids Res..

[22] Francis J.M., Leistritz-Edwards D., Dunn A., Tarr C., Lehman J., Dempsey C., Hamel A., Rayon V., Liu G., Wang Y. (2022). Allelic variation in class I HLA determines CD8 + T cell repertoire shape and cross-reactive memory responses to SARS-CoV-2. Sci. Immunol..

[23] Peng Y., Felce S.L., Dong D., Penkava F., Mentzer A.J., Yao X., Liu G., Yin Z., Chen J.L., Lu Y. (2021). An immunodominant NP105–113-B∗07:02 cytotoxic T cell response controls viral replication and is associated with less severe COVID-19 disease. Nat. Immunol..

[24] Dash P., Fiore-Gartland A.J., Hertz T., Wang G.C., Sharma S., Souquette A., Crawford J.C., Clemens E.B., Nguyen T.H.O., Kedzierska K. (2017). Quantifiable predictive features define epitope-specific T cell receptor repertoires. Nature.

[25] Glanville J., Huang H., Nau A., Hatton O., Wagar L.E., Rubelt F., Ji X., Han A., Krams S.M., Pettus C. (2017). Identifying specificity groups in the T cell receptor repertoire. Nature.

[26] Mayer-Blackwell K., Schattgen S., Cohen-Lavi L., Crawford J.C., Souquette A., Gaevert J.A., Hertz T., Thomas P.G., Bradley P., Fiore-Gartland A. (2021). Tcr meta-clonotypes for biomarker discovery with tcrdist3 enabled identification of public, hla-restricted clusters of sars-cov-2 tcrs. Elife.

[27] Joshi K., de Massy M.R., Ismail M., Reading J.L., Uddin I., Woolston A., Hatipoglu E., Oakes T., Rosenthal R., Peacock T. (2019). Spatial heterogeneity of the T cell receptor repertoire reflects the mutational landscape in lung cancer. Nat. Med..

[28] Emerson R.O., DeWitt W.S., Vignali M., Gravley J., Hu J.K., Osborne E.J., Desmarais C., Klinger M., Carlson C.S., Hansen J.A. (2017). Immunosequencing identifies signatures of cytomegalovirus exposure history and HLA-mediated effects on the T cell repertoire. Nat. Genet..

[29] Murugan A., Mora T., Walczak A.M., Callan C.G. (2012). Statistical inference of the generation probability of T-cell receptors from sequence repertoires. Proc. Natl. Acad. Sci. USA.

[30] Sethna Z., Elhanati Y., Callan C.G., Walczak A.M., Mora T. (2019). OLGA: fast computation of generation probabilities of B- and T-cell receptor amino acid sequences and motifs. Bioinformatics.

[31] de Greef P.C., Oakes T., Gerritsen B., Ismail M., Heather J.M., Hermsen R., Chain B., de Boer R.J. (2020). The naive t-cell receptor repertoire has an extremely broad distribution of clone sizes. Elife.

[32] Bartolo L., Afroz S., Pan Y.-G., Xu R., Williams L., Lin C.-F., Tanes C., Bittinger K., Friedman E.S., Gimotty P.A. (2022). SARS-CoV-2-specific T cells in unexposed adults display broad trafficking potential and cross-react with commensal antigens. Sci. Immunol..

[33] Altmann D.M., Reynolds C.J., Boyton R.J. (2021). SARS-CoV-2 variants: subversion of antibody response and predicted impact on T cell recognition. Cell Rep. Med..

[34] Becerra-Artiles A., Calvo-Calle J.M., Co M., Nanaware P., Cruz J., Weaver G.C., Lu L., Forcini C., Finberg R.W., Moormann A. (2022). Broadly-recognized, cross-reactive SARS-CoV-2 CD4 T cell epitopes are highly conserved across human coronaviruses and presented by common HLA alleles. bioRxiv.

[35] van der Most R.G., Murali-Krishna K., Whitton J.L., Oseroff C., Alexander J., Southwood S., Sidney J., Chesnut R.W., Sette A., Ahmed R. (1998). Identification of Db- and Kb-restricted subdominant cytotoxic T-cell responses in lymphocytic choriomeningitis virus-infected mice. Virology.

[36] Gairin J.E., Mazarguil H., Hudrisier D., Oldstone M.B. (1995). Optimal lymphocytic choriomeningitis virus sequences restricted by H-2Db major histocompatibility complex class I molecules and presented to cytotoxic T lymphocytes. J. Virol..

[37] Dow C., Oseroff C., Peters B., Nance-Sotelo C., Sidney J., Buchmeier M., Sette A., Mothé B.R. (2008). Lymphocytic choriomeningitis virus infection yields overlapping CD4+ and CD8+ T-cell responses. J. Virol..

[38] Minervina A.A., Pogorelyy M.v., Kirk A.M., Crawford J.C., Allen E.K., Chou C.-H., Mettelman R.C., Allison K.J., Lin C.-Y., Brice D.C. (2022). SARS-CoV-2 antigen exposure history shapes phenotypes and specificity of memory CD8+ T cells. Nat. Immunol..

[39] Reynolds C.J., Swadling L., Gibbons J.M., Pade C., Jensen M.P., Diniz M.O., Schmidt N.M., Butler D.K., Amin O.E., Bailey S.N.L. (2020). Discordant neutralizing antibody and T cell responses in asymptomatic and mild SARS-CoV-2 infection. Sci. Immunol..

[40] Bains I., Antia R., Callard R., Yates A.J. (2009). Quantifying the development of the peripheral naive CD4+ T-cell pool in humans. Blood.

[41] Pogorelyy M.V., Minervina A.A., Touzel M.P., Sycheva A.L., Komech E.A., Kovalenko E.I., Karganova G.G., Egorov E.S., Komkov A.Y., Chudakov D.M. (2018). Precise tracking of vaccine-responding T cell clones reveals convergent and personalized response in identical twins. Proc. Natl. Acad. Sci. USA.

[42] DeWitt W.S., Emerson R.O., Lindau P., Vignali M., Snyder T.M., Desmarais C., Sanders C., Utsugi H., Warren E.H., McElrath J. (2015). Dynamics of the cytotoxic T cell response to a model of acute viral infection. J. Virol..

[43] Rydyznski Moderbacher C., Ramirez S.I., Dan J.M., Grifoni A., Hastie K.M., Weiskopf D., Belanger S., Abbott R.K., Kim C., Choi J. (2020). Antigen-specific adaptive immunity to SARS-CoV-2 in acute COVID-19 and associations with age and disease severity. Cell.

[44] Qi Q., Liu Y., Cheng Y., Glanville J., Zhang D., Lee J.Y., Olshen R.A., Weyand C.M., Boyd S.D., Goronzy J.J. (2014). Diversity and clonal selection in the human T-cell repertoire. Proc. Natl. Acad. Sci. USA.

[45] Zinkernagel R.M. (2002). Lymphocytic choriomeningitis virus and immunology. Curr. Top. Microbiol. Immunol..

[46] Oakes T., Heather J.M., Best K., Byng-Maddick R., Husovsky C., Ismail M., Joshi K., Maxwell G., Noursadeghi M., Riddell N. (2017). Quantitative characterization of the T cell receptor repertoire of naïve and memory subsets using an integrated experimental and computational pipeline which is robust, economical, and versatile. Front. Immunol..

[47] Uddin I., Joshi K., Oakes T., Heather J.M., Swanton C., Chain B. (2019). An economical, quantitative, and robust protocol for high-throughput T cell receptor sequencing from tumor or blood. Methods Enzymol..

[48] Peacock T., Heather J.M., Ronel T., Chain B. (2021). Decombinator V4: an improved AIRR compliant-software package for T-cell receptor sequence annotation?. Bioinformatics.

[49] R Core Team (2021). https://www.R-project.org.

[50] Agarwala R., Barrett T., Beck J., Benson D.A., Bollin C., Bolton E., Bryant S.H., Canese K., Church D.M., NCBI Resource Coordinators (2014). Database resources of the national center for biotechnology information. Nucleic Acids Res..

[51] Astbury S., Reynolds C.J., Butler D.K., Muñoz-Sandoval D.C., Lin K.M., Pieper F.P., Otter A., Kouraki A., Cusin L., Nightingale J. (2022). HLA-DR polymorphism in SARS-CoV-2 infection and susceptibility to symptomatic COVID-19. Immunology.

[52] Chang C.C., Chow C.C., Tellier L.C., Vattikuti S., Purcell S.M., Lee J.J. (2015). Second-generation PLINK: rising to the challenge of larger and richer datasets. GigaScience.

[53] Zheng X., Shen J., Cox C., Wakefield J.C., Ehm M.G., Nelson M.R., Weir B.S. (2014). HIBAG--HLA genotype imputation with attribute bagging. Pharmacogenomics J..

[54] Bolotin D.A., Poslavsky S., Mitrophanov I., Shugay M., Mamedov I.Z., Putintseva E. v, Chudakov D.M. (2015). MiXCR: software for comprehensive adaptive immunity profiling. Nat. Methods.

[55] Mark M., Reich-Zeliger S., Greenstein E., Reshef D., Madi A., Chain B., Friedman N. (2021). Antigen experience relaxes the organisational structure of the T cell receptor repertoire. bioRxiv.

[56] Karatzoglou A., Smola A., Hornik K., Zeileis A. (2004). **Kernlab** - an *S4* package for kernel methods in *R*. J. Stat. Softw..

[57] Csardi G., Nepusz T. (2006).

[58] Tan C.C.S., Owen C.J., Tham C.Y.L., Bertoletti A., van Dorp L., Balloux F. (2021). Pre-existing T cell-mediated cross-reactivity to SARS-CoV-2 cannot solely be explained by prior exposure to endemic human coronaviruses. Infect. Genet. Evol..

[59] Elbe S., Buckland-Merrett G. (2017). Data, disease and diplomacy: GISAID’s innovative contribution to global health. Glob. Chall..

[60] Shu Y., McCauley J. (2017). GISAID: global initiative on sharing all influenza data – from vision to reality. Euro Surveill..

[61] Katoh K., Misawa K., Kuma K.I., Miyata T. (2002). MAFFT: a novel method for rapid multiple sequence alignment based on fast Fourier transform. Nucleic Acids Res..

[62] Huddleston J., Hadfield J., Sibley T.R., Lee J., Fay K., Ilcisin M., Harkins E., Bedford T., Neher R.A., Hodcroft E.B. (2021). Augur: a bioinformatics toolkit for phylogenetic analyses of human pathogens. J. Open Source Softw..

[63] Paradis E., Claude J., Strimmer K. (2004). APE: analyses of phylogenetics and evolution in R language. Bioinformatics.

