# Supplementary material for: Large clones of pre-existing T cells drive early immunity against SARS-COV-2 and LCMV infection
Source: iScience. 2023 May 22;26(6):106937. doi: 10.1016/j.isci.2023.106937 (PMC10201888; doi:10.1016/j.isci.2023.106937)
Supplement: Document S1. Figures S1–S15 [file mmc1.pdf]

iScience, Volume *26*

Supplemental information

Large clones of pre-existing T cells drive early immunity against SARS-COV-2 and LCMV infection

Martina Milighetti, Yanchun Peng, Cedric Tan, Michal Mark, Gayathri Nageswaran, Suzanne Byrne, Tahel Ronel, Tom Peacock, Andreas Mayer, Aneesh Chandran, Joshua Rosenheim, Matthew Whelan, Xuan Yao, Guihai Liu, Suet Ling Felce, Tao Dong, Alexander J. Mentzer, Julian C. Knight, Francois Balloux, Erez Greenstein, Shlomit Reich-Zeliger, Corinna Pade, Joseph M. Gibbons, Amanda Semper, Tim Brooks, Ashley Otter, Daniel M. Altmann, Rosemary J. Boyton, Mala K. Maini, Aine McKnight, Charlotte Manisty, Thomas A. Treibel, James C. Moon, COVIDsortium Investigators, Mahdad Noursadeghi, and Benny Chain

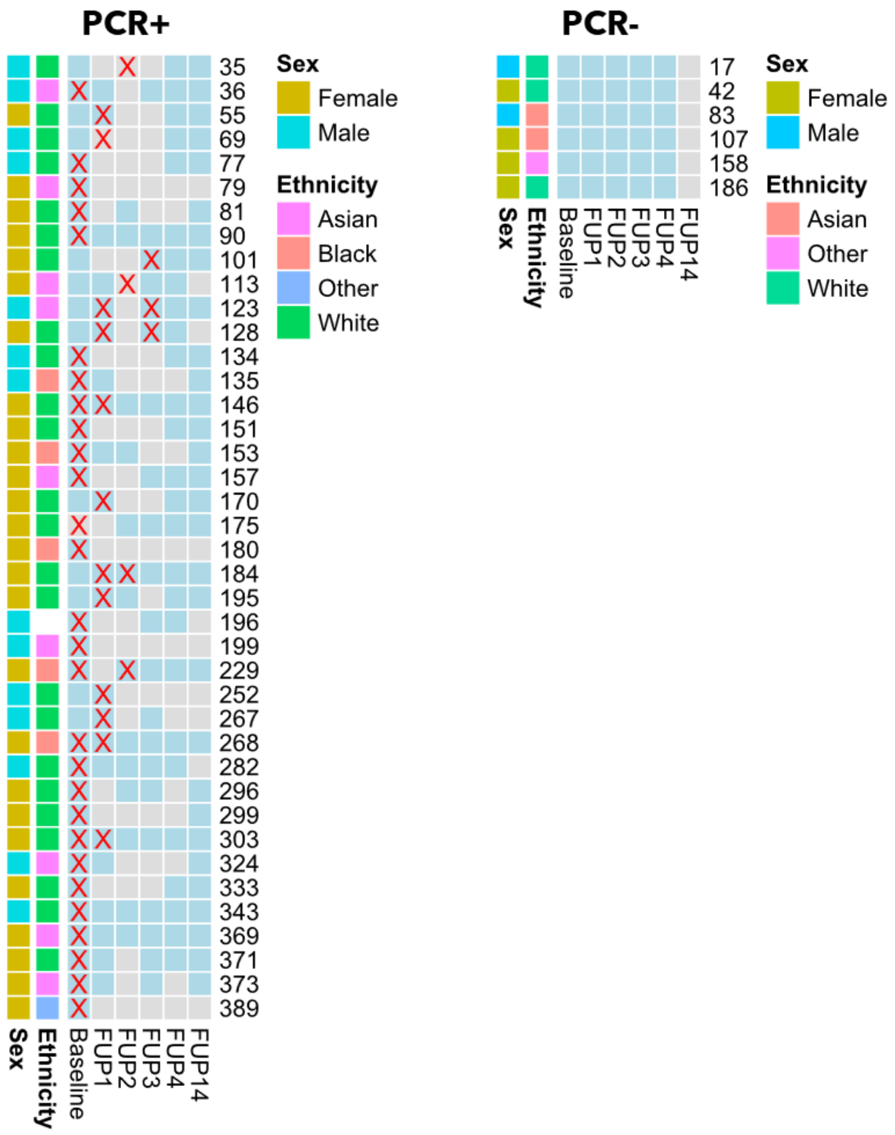

Figure S1: Summary of samples from baseline, and follow-up (FUP) from the COVIDsortium study (related to Figure 1). Each row represents one individual, and the anonymized study ID is shown to the right of the row. The samples for which RNA was available are shown in light blue. The red X shows the time of first PCR+ test. Basic demographics are shown for each individual.

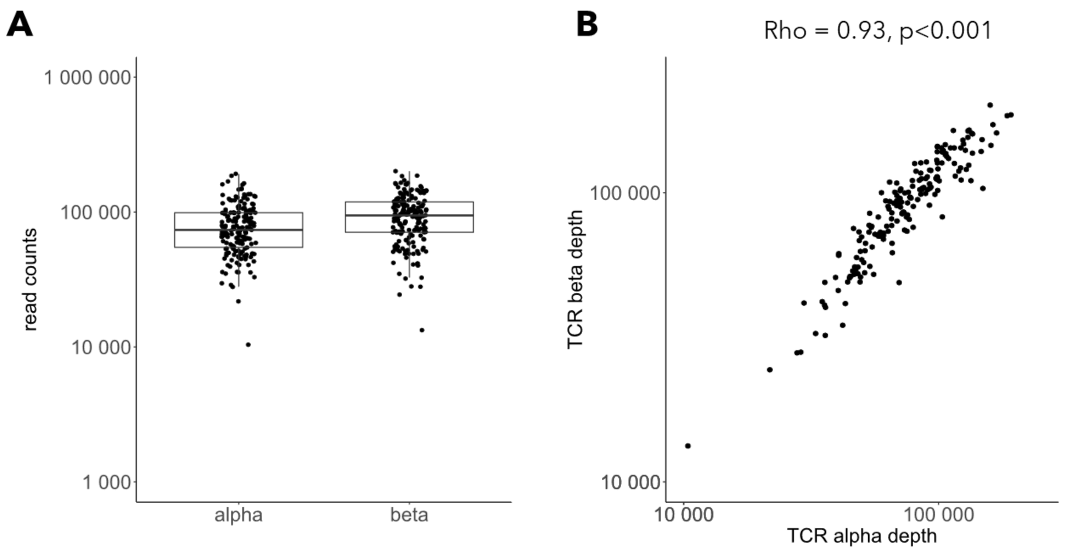

Figure S2: TCR sequencing depth per sample (related to Figure 1). A. Total number of TCRs sequenced per sample. The boxplots show median, interquartile (box) and 95% (whiskers) range. **B.** Total number of beta versus total number of alpha per sample and Spearman correlation test results.

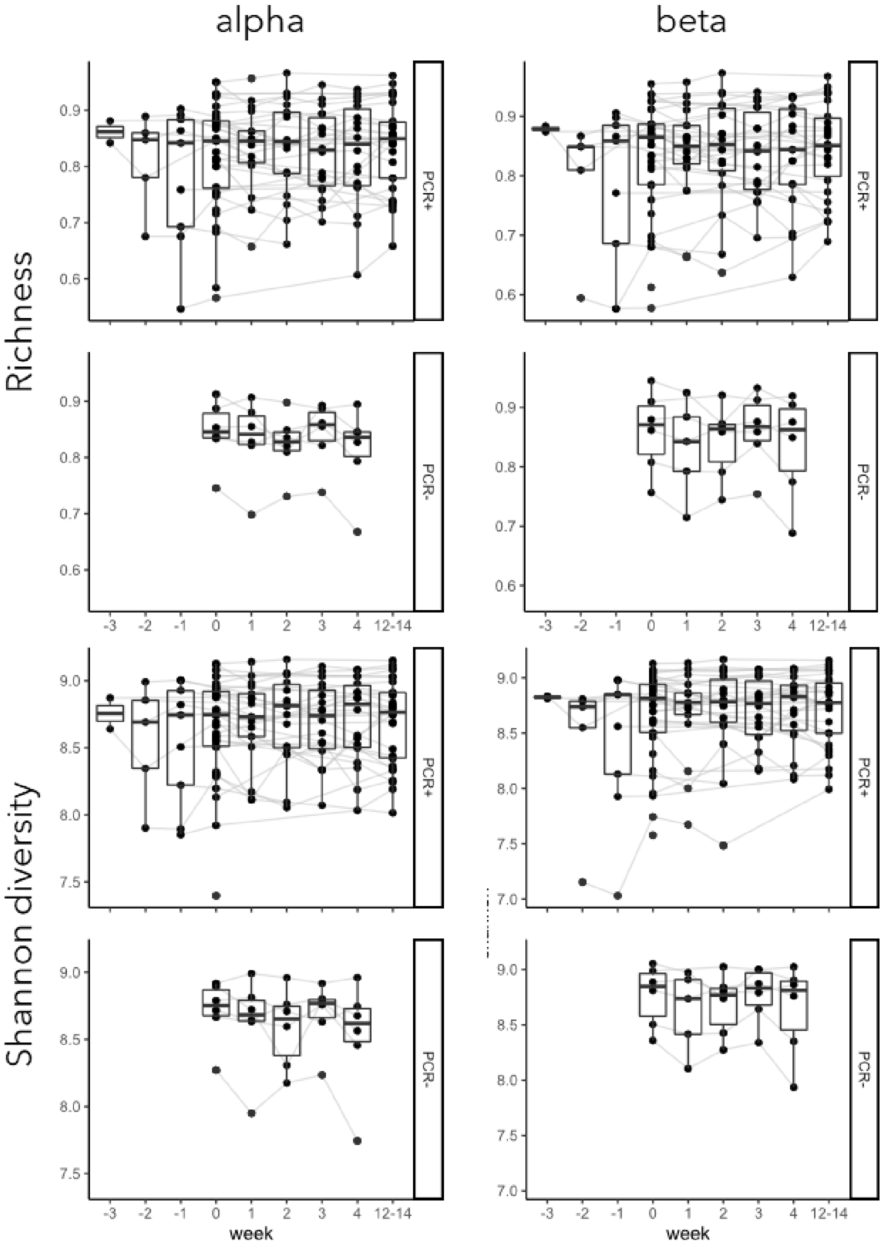

Figure S3: Richness and Shannon diversity of whole TCR repertoires at each time point (related to Figure 1). Richness and diversity are calculated on whole TCR repertoires, without selecting for expanded TCRs. For PCR+ individuals, the x-axis is rescaled relative to the week at which they first became PCR+ (this is week 0). For PCR- individuals the weeks correspond to baseline and subsequent follow-ups at weeks 1-4. The boxplots show median, interquartile (box) and 95% (whiskers) range.

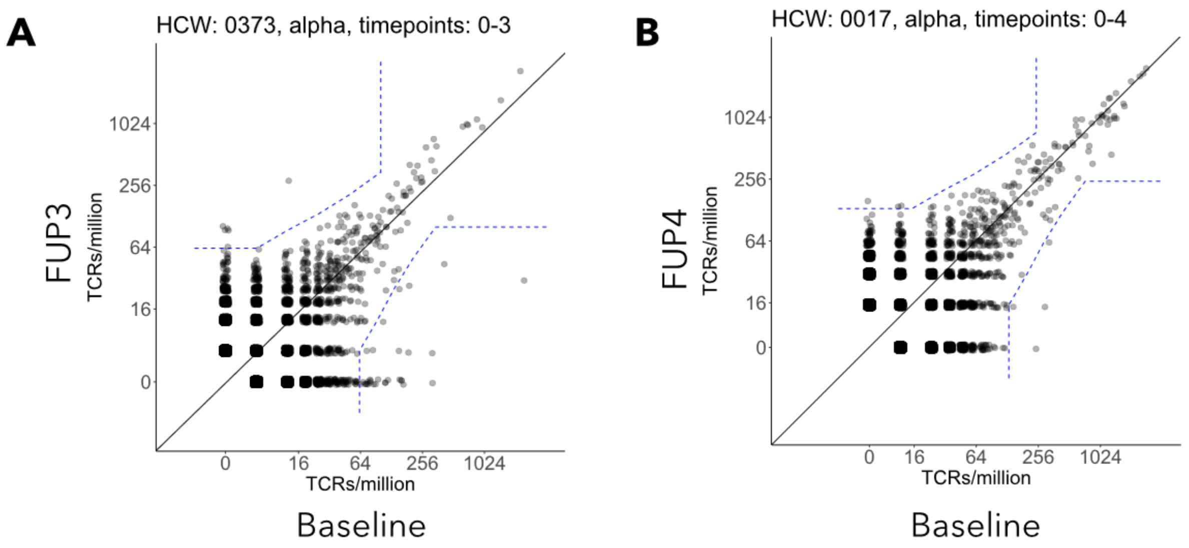

Figure S4: Examples of contracting TCRs in individual PCR+ at baseline, and constant TCRs in PCR- individual (related to Figure 1). A. An example of a pairwise comparison showing TCRs contracting between baseline and FUP3 timepoints. The individual (ID 373) was PCR+ at baseline. Each point is an individual TCR sequence, and the plot shows abundance at FUP3 versus abundance at Baseline. All abundances are normalized to number of TCRs per million. The dashed blue line indicates the significance threshold calculated as described in M&M. All TCRs which fall outside the dashed line are considered as expanded (or contracted). **B.** As in A, comparing repertoires at baseline and FUP4 in a control individual (ID 17) who did not become PCR+ or seroconvert during the study. Note the small number of expanding and contracting TCRs.

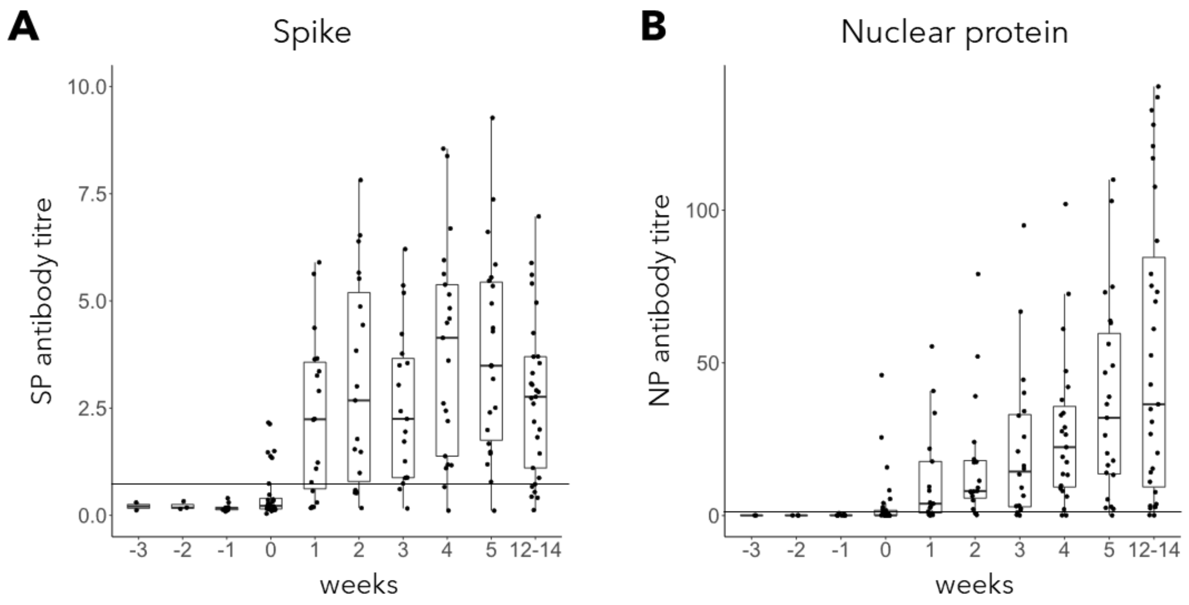

Figure S5: The kinetics of anti-SARS-COV-2 antibody in serum of the HCW who became PCR+ (related to Figure 1). The y-axis is an arbitrary scale of antibody titre. The limits of detection are shown by a horizontal line. The x-axis is rescaled relative to the week at which they first became PCR+ (this is week 0).

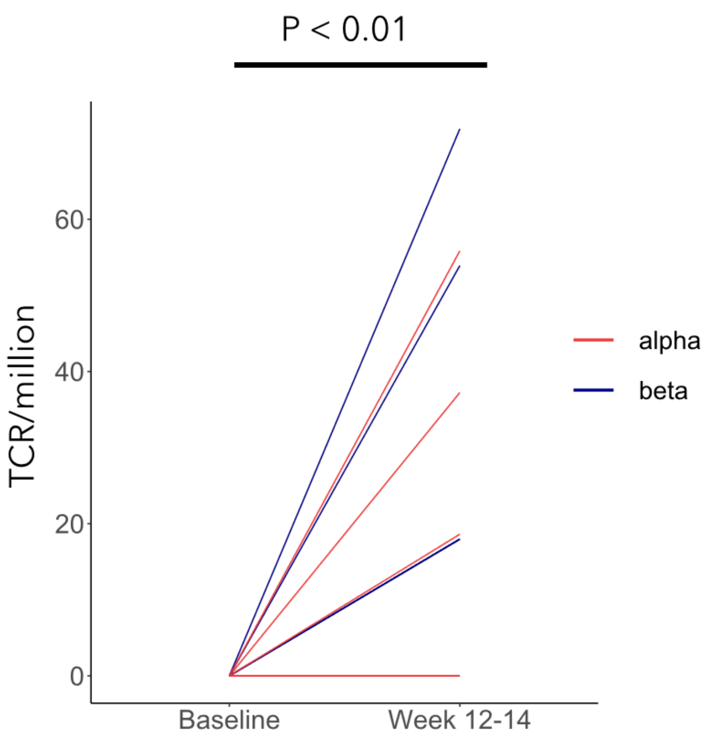

Figure S6: Comparison of TCR abundance at baseline (pre-PCR+, week -3) and week 14 for HCW 101 (related to Figure 1). TCRalpha is shown in red, TCRbeta in blue. P-value calculated with Kruskal-Wallis H test.

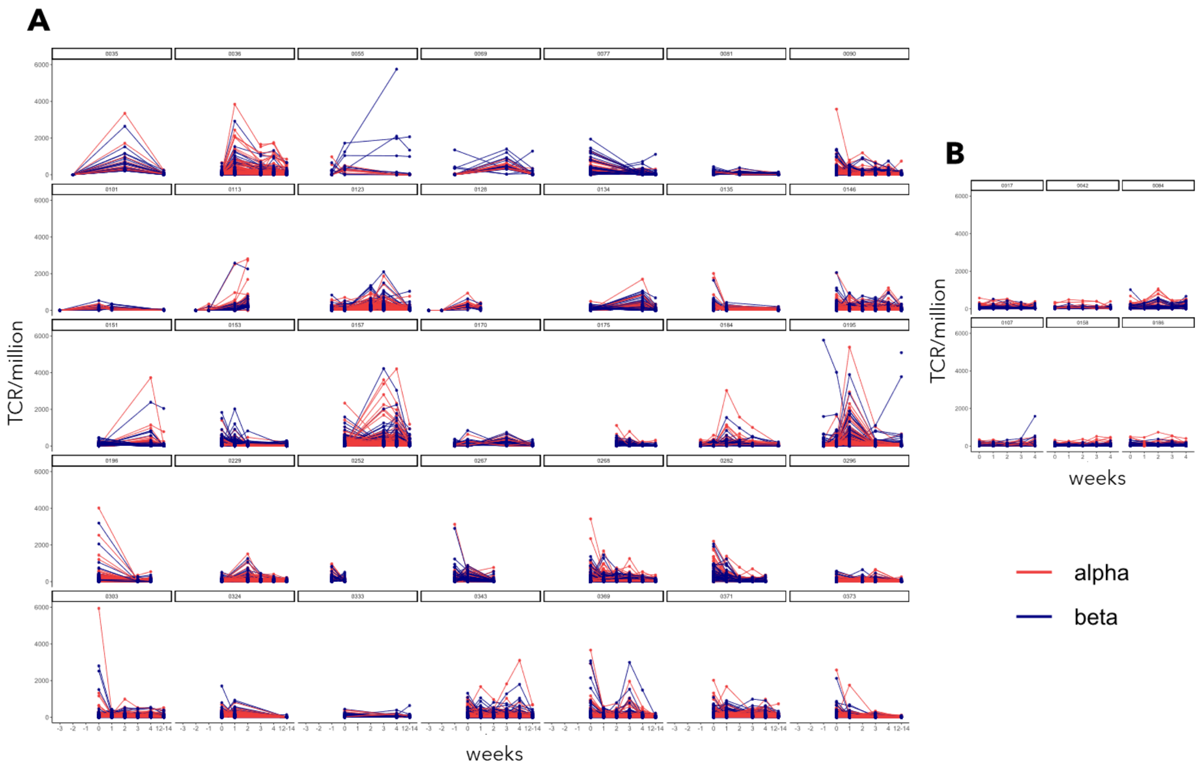

Figure S7: Kinetics of changing TCRs in individual repertoires (related to Fig 1). A. In each PCR+ HCW. The x-axis is rescaled relative to the week at which they first became PCR+ (this is week 0). **B.** In each PCR- control (same scale for comparison).

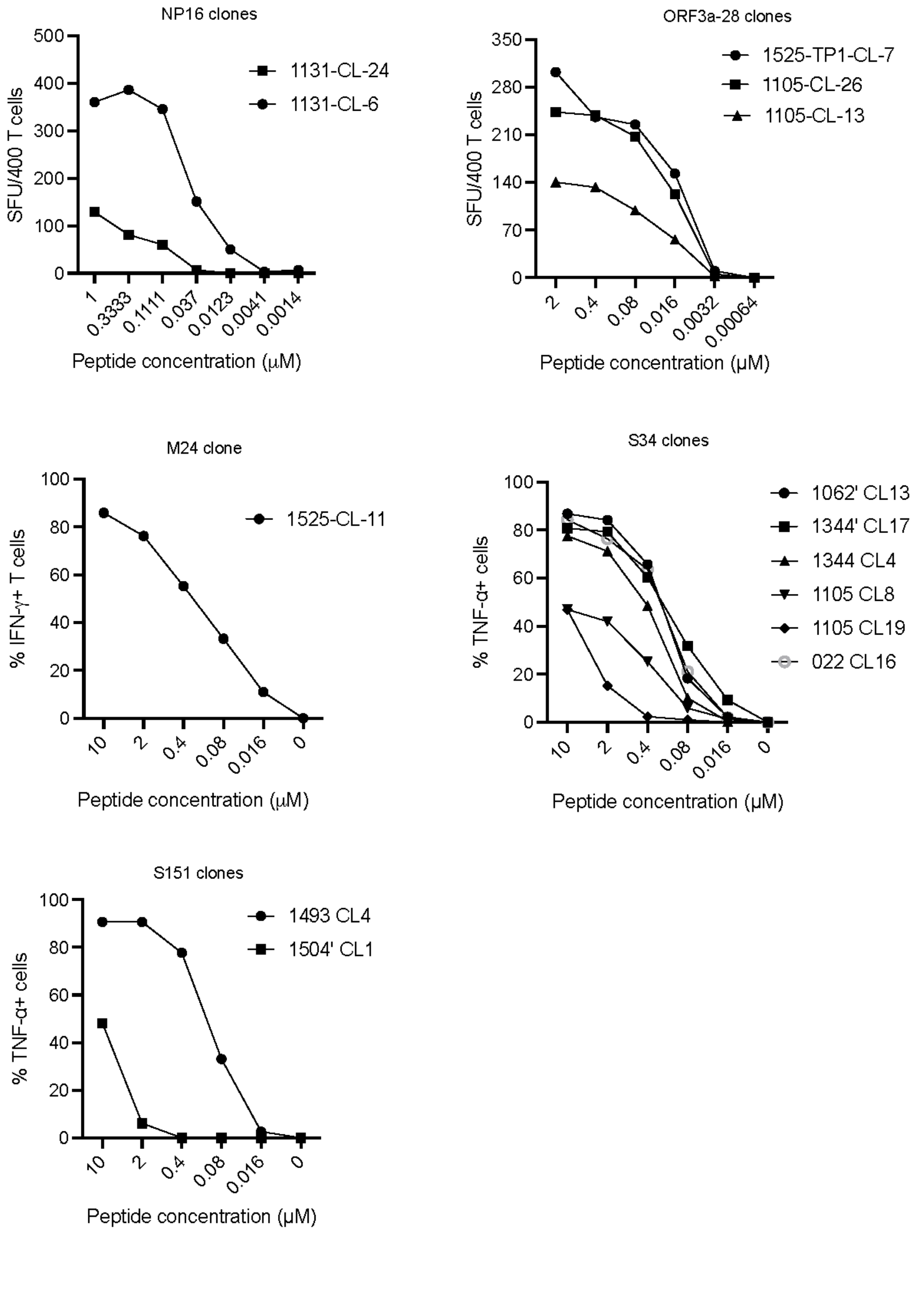

Figure S8: Evaluation of antigen sensitivity of SARS-CoV-2 specific T cells (related to Figure 2). SARS-CoV-2 specific T cell clones were generated as previously described https://doi.org/10.3389/fimmu.2015.00287. T cell clones were co-cultured with target cells loaded with peptide at titrated concentration and cytokine production of each T cell clone was assessed by IFN-γ ELISpot or intracellular cytokine staining.

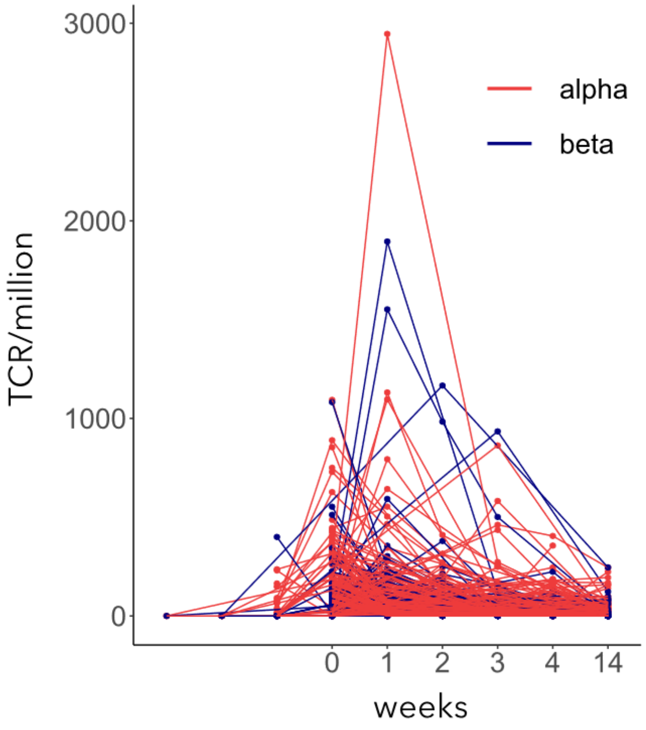

Figure S9: The time course for TCRs which are both expanded and annotated as recognising SARS-COV-2 antigens (related to Figure 2). Each line is a TCR which is expanded and which is found in at least one of the annotated sets. The x-axis is rescaled relative to the week at which they first became PCR+ (this is week 0).

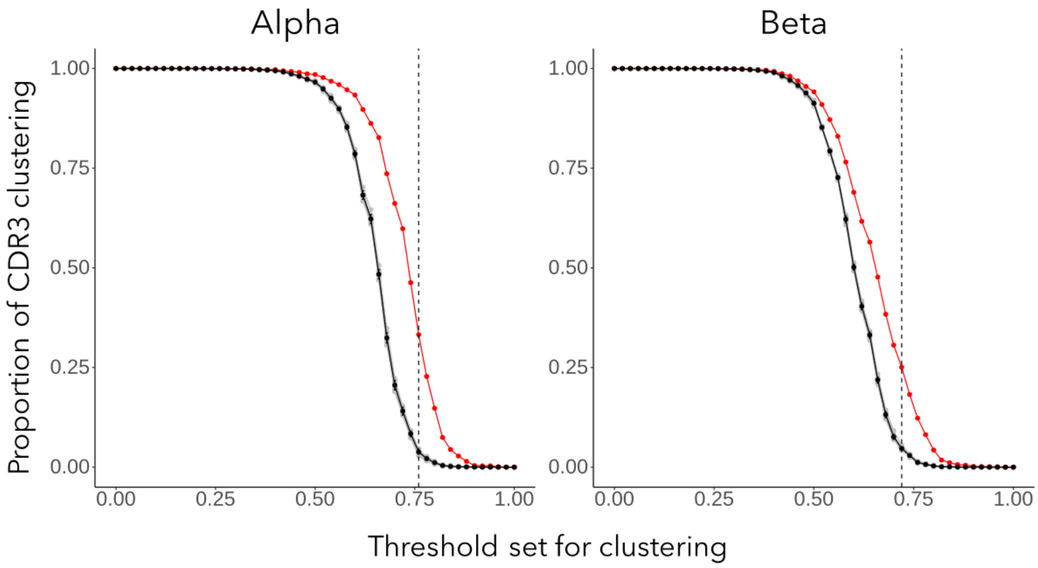

Figure S10: The proportion of TCRalpha and TCRbeta within a cluster (cluster size > 1) at different similarity thresholds (related to Figure 2). The red line shows the SARS-COV-2 expanded TCRs; the black line shows the mean of 10 sets of control (non-expanded) TCRs. The vertical dotted line shows the threshold used in the plots shown in Fig 2F.

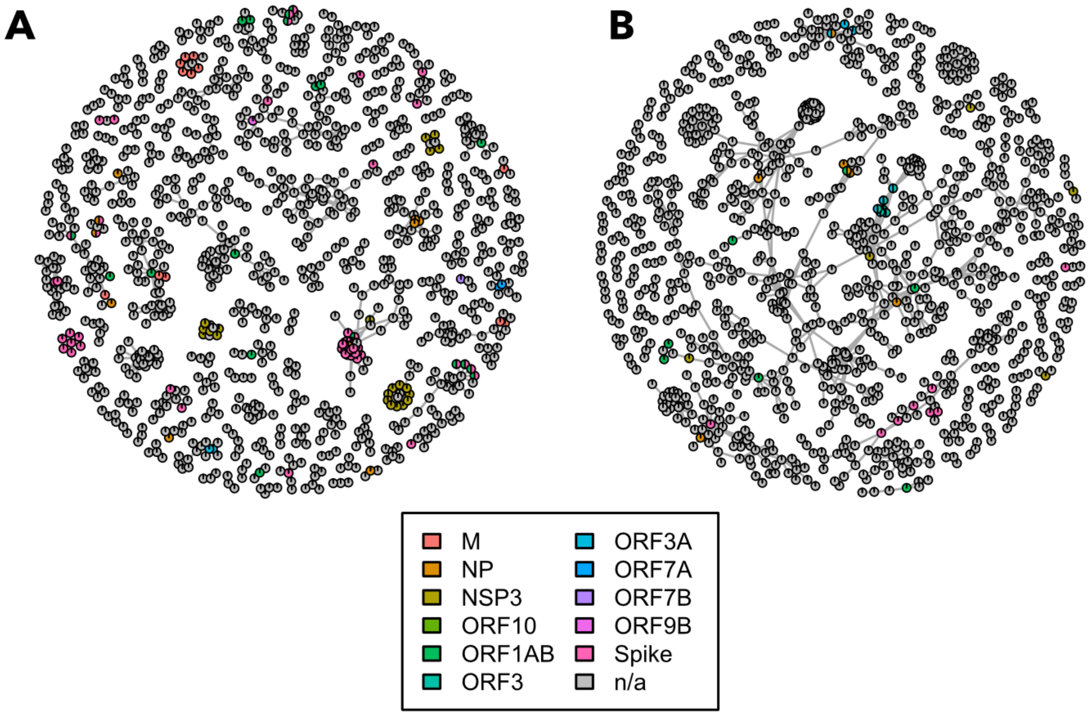

Figure S11: Clustering with annotated sequences (related to Figure 2). Clustered expanded TCRalpha (**A**) or TCRbeta (**B**) sequences (clusters with greater than 3 nodes are shown) coloured according to target antigen. The grey nodes represent unannotated TCRs for which the antigen is not known.

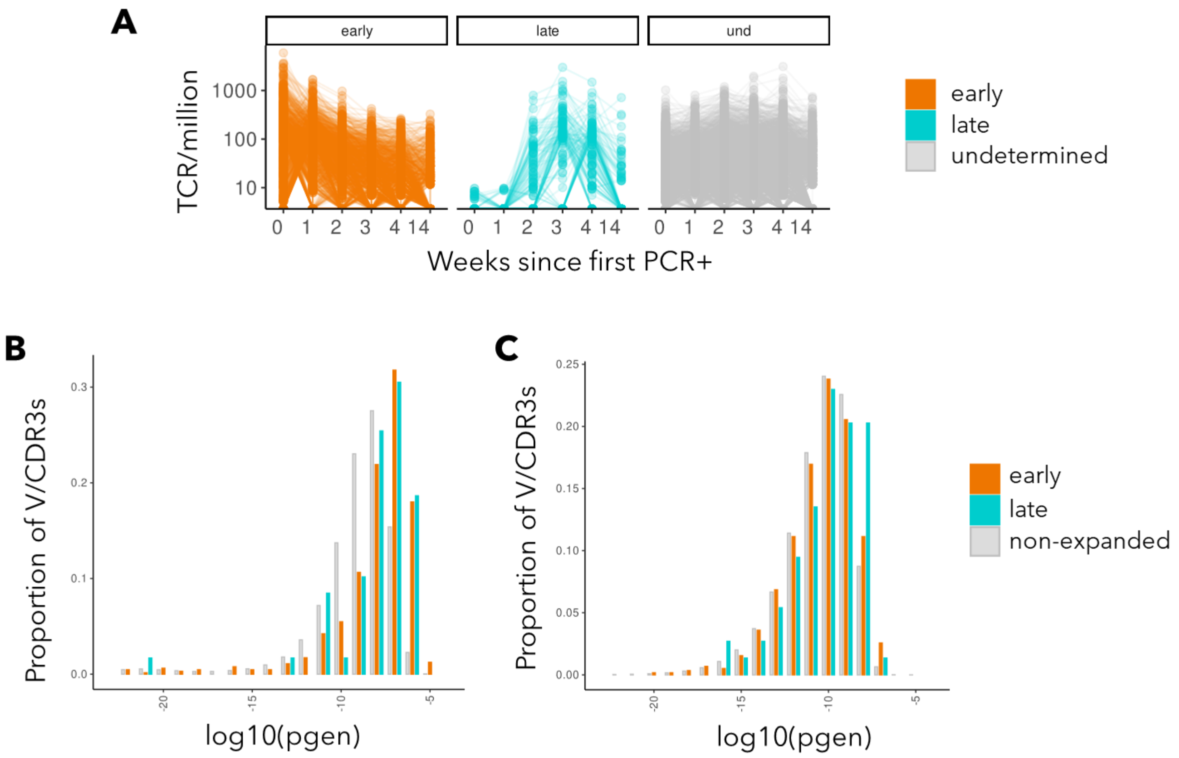

Figure S12: Studying early and late expanding TCRs (related to Figure 3). A. Definition of TCRs as early (orange) or late (cyan) across all HCW samples. In grey, TCRs that we could not classify as early or late. **B.** P(gen) calculated on V and CDR3 alpha amino acid sequence. **C.** P(gen) calculated on V and CDR3 beta amino acid sequence.

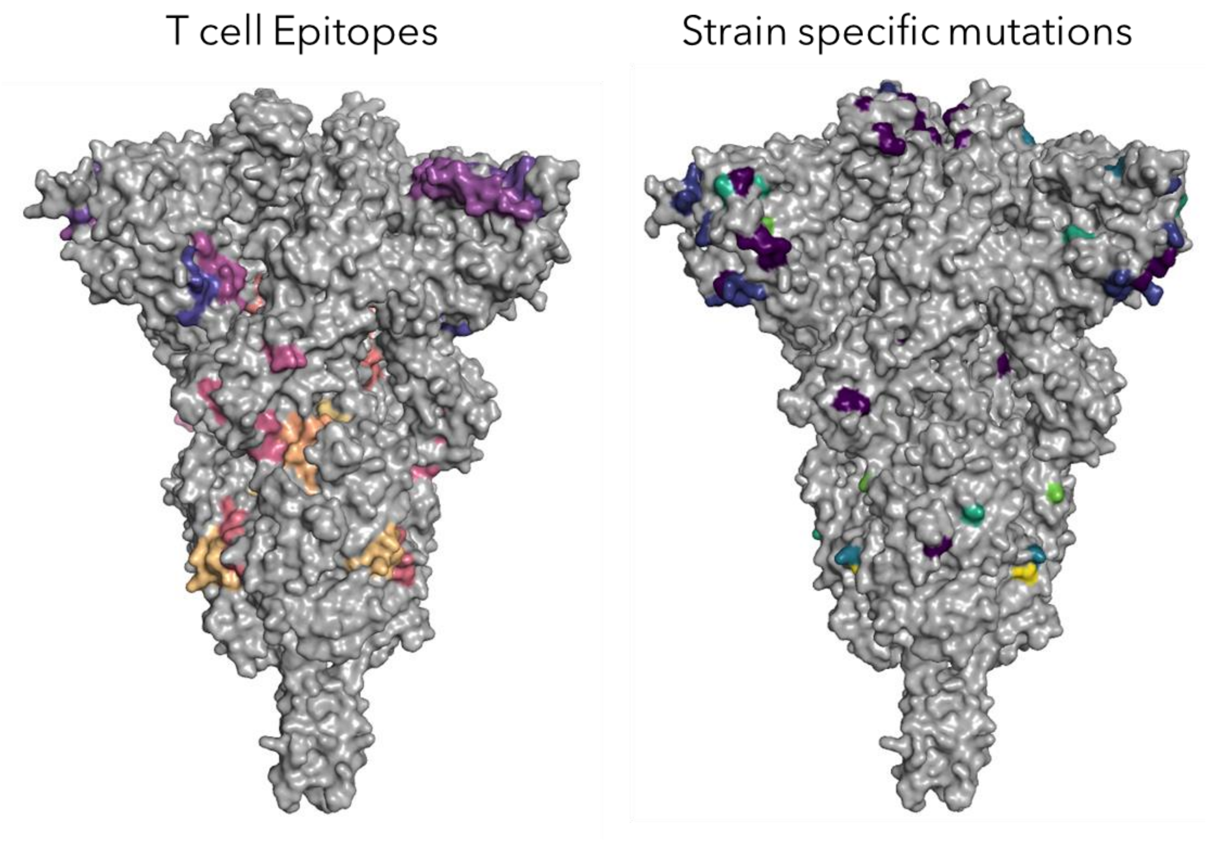

Figure S13: Location of defined spike T cell epitopes and strain-specific mutations within the spike protein structure (related to Figure 4).

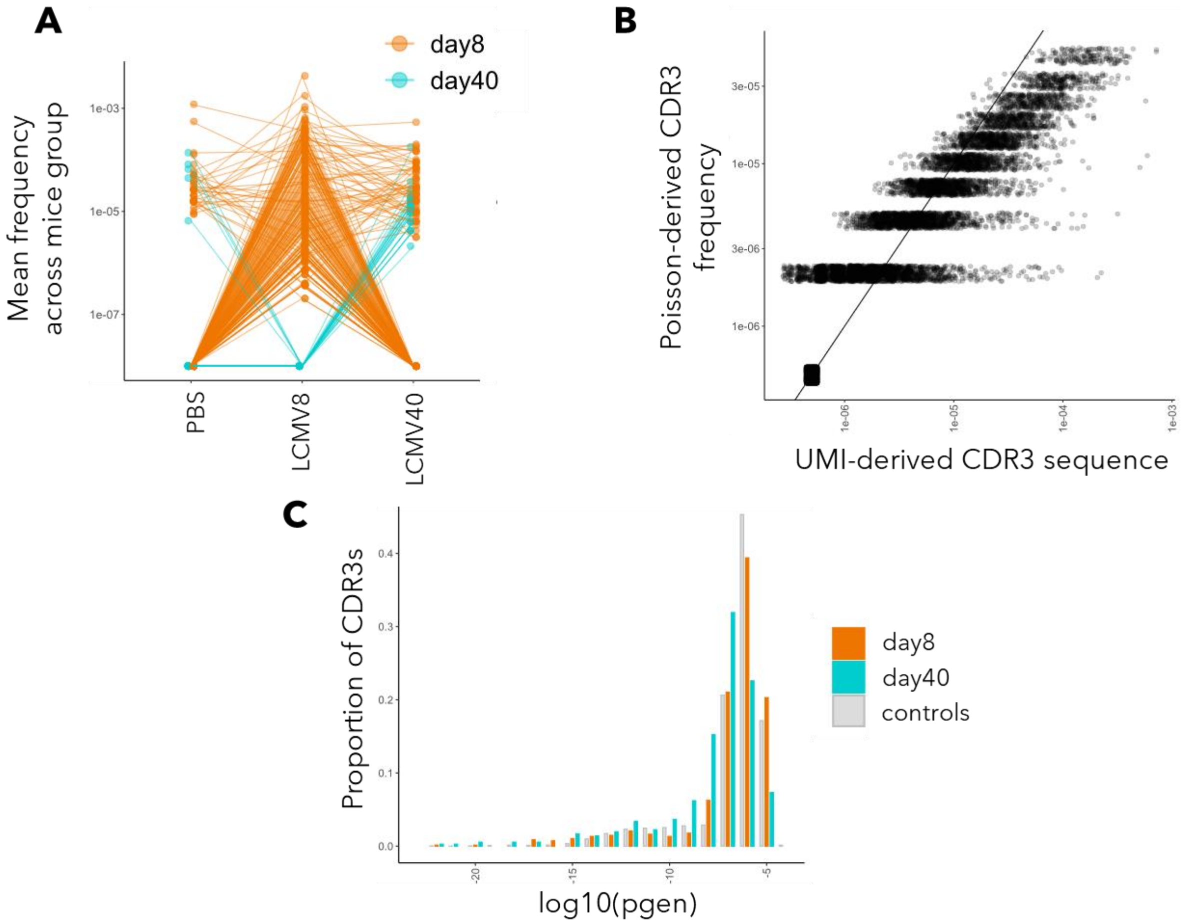

Figure S14: Studying early and late expanding TCRs in LCMV infection (related to Figure 5). A. Definition of effectors as expanded day 8 (orange) or expanded day 40 (cyan). The average frequency of each CDR3 across the 3 (or 4) mice at each timepoint is shown. **B.** The naïve frequency of each CDR3 inferred with a method based on Poisson statistics and calculated from a quantitative sequencing protocol correlate with each other (Pearson R = 0.78, p-value < 2.2e-16) **C.** P(gen) calculated on CDR3 aa sequence.

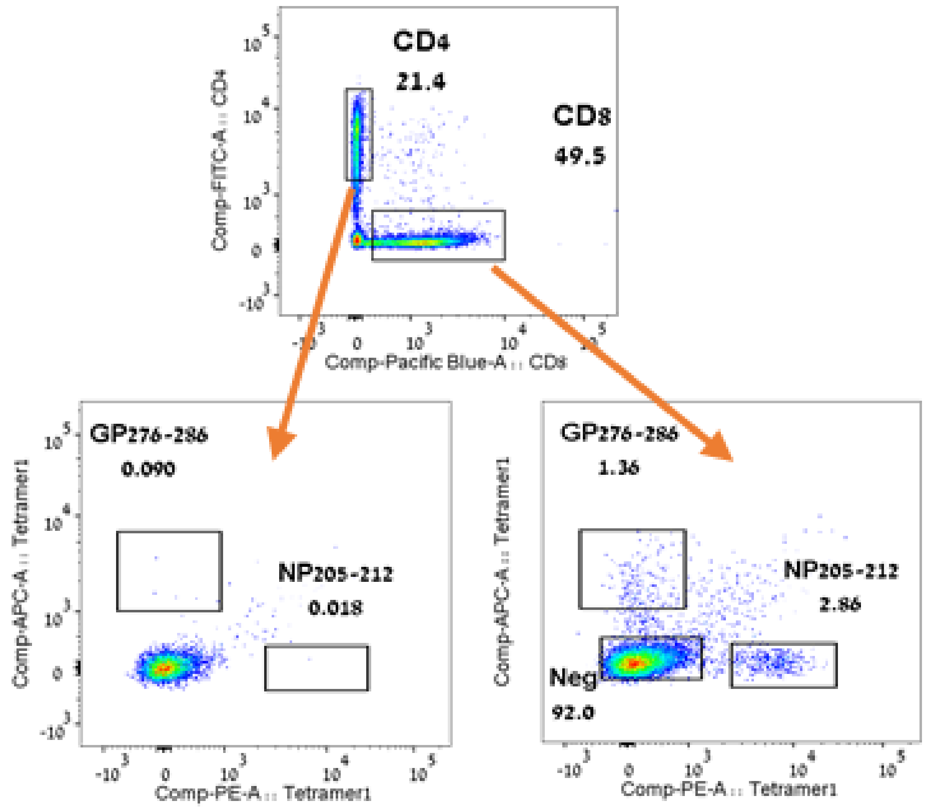

Figure S15: Representative sort plot for isolation of CD8+ LCMV tetramer positive cells (related to Figure 5).
